# Cooperative care influences genome-wide levels of DNA methylation in nestling chestnut-crowned babblers

**DOI:** 10.1098/rstb.2025.0014

**Published:** 2026-07-23

**Authors:** Andrea L. Liebl, Susan C. Anderson, Andrew F. Russell, Jeff S. Wesner, Jason L. Petersen

**Affiliations:** ^1^ Department of Biology, University of South Dakota, Vermillion, SD, USA; ^2^ Centre for Ecology & Conservation, School of Biosciences, University of Exeter Cornwall Campus, Penryn, Cornwall, UK; ^3^ Avera McKennan Hospital and University Health Center, Sioux Falls, SD, USA

**Keywords:** carers, cooperative breeding, early life, epigenetics, developmental environment, helpers

## Abstract

Carers in cooperatively breeding vertebrates increase food acquisition for offspring; however, they also impact the developmental social environment. One means of linking early-life environments, such as nutrition and social structure, with later-life phenotypes is DNA methylation. Here, using whole-genome methylation sequencing, we measured how additional carers influence DNA methylation in nestlings of the cooperatively breeding chestnut-crowned babbler (*Pomatostomus ruficeps*). A comparison of nestlings raised by their parents (two carers) and those raised by parents plus additional helpers (‘three plus’ carers; mean = 4.3 ± 1.4 s.d.) revealed that additional care is associated with genome-wide differences in DNA methylation. Overall, 570 cytosine-phosphate-guanine sites from the regulatory regions of 487 genes were differentially methylated, with 85% being more methylated in nestlings reared by groups. Specifially, sites associated with genes that are integral for metabolism, growth, the regulation and promotion of sociality, the ability to cope with stressors, and cell communication were differentially methylated between the groups. Furthermore, gene ontology-term analysis revealed that differentially methylated sites were over-represented in multiple pathways, including those important for protein binding, metabolism and cell-to-cell and environment-to-cell communication. Our results suggest that the effects of being reared by groups as opposed to pairs in cooperative breeders can extend beyond those typically attributed to nutritional benefits and that these effects are molecularly mediated.

This article is part of the theme issue ‘Ecological epigenetics at the intersection of behaviour and life history variation in non-model animals’.

## 1. Introduction

Understanding the evolution of cooperative breeding requires an understanding of the downstream effects that helpers have on recipient offspring. Research has demonstrated that helpers not only impact offspring survival before independence by providing additional food during development [1] but also their survival, dispersal, and breeding probabilities post-independence [2–5]. The mechanism mediating such post-independence effects is typically suggested to be the result of long-term benefits of increased food, leading to better body condition later in life. For example, in cooperatively breeding long-tailed tits (*Aegithalos caudatus*), offspring reared by more helpers are heavier at fledging and are more likely to survive to the following year [4]. Similarly, in meerkats (*Suricata suricatta*), changing the ratio of offspring to adults influences offspring weight gain; supplemental feeding confirms the importance of food for long-term survival [2] as well as the probabilities of dispersal and reproduction as an adult [3]. However, although increased survival is often assumed to be a result of provisioning, the direct impact of helpers is not always clear: in addition to nutritional benefits, each provisioning effort also results in, by definition, a social interaction between offspring and the provisioning adult. Thus, having adult carers at the nest also provides additional information, such as group size and social structure, to developing nestlings about the social interactions they will experience following independence. Processing such information (e.g. through provisioning visits or recognition of individual calls [6,7] is likely to be beneficial postfledging, as it has implications for how individuals respond physiologically and behaviourally to social interactions. However, we know little about how social information is transmitted to offspring during development among cooperative breeders. Addressing this shortcoming will provide a more holistic picture of the effect of helpers on offspring and the underlying mechanisms that are influenced by differences in adult care. This will provide new insights into long-standing questions regarding the evolution and within-lifetime development of cooperative versus reproductive behaviour.

In addition to nutritional effects, social environments during rearing can also impact social behaviours and the ability to cope with stressors later in life [8–10]. For instance, jumping spiders (*Marpissa muscosa*) reared in social groups performed better in subsequent associative learning and social tasks than those reared alone [11]. Similarly, offspring of the cooperative cichlid (*Neolamprologus pulcher*) raised in more complex social groups with older siblings show greater social competence across a range of social situations later in life [9,12,13]. In coping with stressors, the presence of helpers is associated with greater capacity to mount and regulate stress responses in postweaned offspring of alpine marmots (*Marmota marmota*) [14]. Furthermore, increased socialization in developing white-faced marmosets (*Callithrix geoffryoi*) leads to decreased basal glucocorticoids later in life [15]. Although some of these results may not be independent of nutrition, they suggest that developmental social environments, including the presence of helpers, probably have additional impacts on traits that contribute to offspring success later in life.

One molecular mechanism that is expected to mediate connections between early-life social environments and later-life phenotypes is epigenetics, particularly DNA methylation. Many epigenetic changes are derived from interactions between the environment and the genome, allowing exposure to a single environment to induce an epigenetic change that can dictate a phenotypic trajectory throughout life [16–21]. As such, it is expected that epigenetic changes respond to differences in the environment as a mechanism to ‘prime’ phenotypes to the environment. Importantly, early-life social environments influence DNA methylation, including genes linked to growth, metabolism, the regulation and promotion of sociality, and the stress response. For example, one classic example of the effects of early-life environments on epigenetically derived phenotypes is related to maternal care in rats. Specifically, low levels of maternal care lead to an increase in DNA methylation of particular loci in the promoter of offspring glucocorticoid receptors (*NR3C1*), which results in reduced regulation of the hypothalamic–pituitary–adrenal (HPA) axis and a reduced capacity to cope with stressors, increased anxiety, and reduced parental care in adult offspring [20]. In addition, reduced maternal care has been linked to variation in the DNA methylation of genes important for regulating and promoting social behaviour, such as *BDNF* and the oxytocin receptor, in humans and in rats [22,23]. Finally, increased maternal care and social connectedness in juvenile spotted hyenas (*Crocuta crocuta*) were related to global changes in DNA methylation that corresponded with lower faecal glucocorticoid metabolite concentrations [24]. Nevertheless, it is currently unclear whether being reared by additional carers in cooperative breeding systems influences epigenetic marks and, if so, which pathways are affected.

To assess how the presence of helpers influences offspring DNA methylation during development, we used natural variation in carer number in conjunction with a cross-fostering experiment in the cooperatively breeding chestnut-crowned babbler (*Pomatostomus ruficeps*). Chestnut-crowned babblers are a 50 g passerine endemic to arid and semi-arid southeastern Australia. This non-territorial bird breeds in pairs and cooperative groups with varying numbers of helpers (typically male and often related to one or both of the parents) who care for offspring by provisioning [25]. In this species, helpers augment the rate at which nestlings receive food during development in a largely additive manner such that nestlings with more helpers receive more food during the developmental period [26,27]. Increased food delivery has positive effects on nestling survival until fledging (approximately 24 days post-hatching) [28–30], indicating that helpers add nutritional benefits for offspring as they do in other species (see above). In addition to increased food, however, chestnut-crowned babbler social group size also has implications for predator avoidance [31], foraging strategy [32], and metabolism [33]; thus, other factors related to helpers may influence molecular signals derived during development and subsequent phenotypes. Following breeding, breeding units join larger social groups of 3–27 adults (mean = 11) during the non-breeding season [34]. Although the tendency for breeding units to coalesce postbreeding means that breeding unit size is not a direct representation of social group size after fledging, breeding group size during development strongly predicts relatedness and genetic structure, which dictates the competitive-cooperative landscape in groups for fledglings [35,36]. Previous work in this system has indicated that epigenetic markers derived before fledging are associated with subsequent dispersal decisions, indicating links among the rearing environment, epigenetic patterns, and post-developmental behaviour [37].

Here, using a cross-fostering study to control for genetic effects, we investigated how the presence of helpers influenced whole-genome DNA methylation of erythrocytes before fledging (approximately 16 days). Given the ability of DNA methylation to prime phenotypes, we predicted that the number of carers at the nest would influence methylation throughout the genome, particularly in the functional regions of genes related to metabolism, growth, stress resistance, and the regulation and promotion of sociality. First, we confirmed the differences in provisioning between nestlings reared by pairs or groups. Second, we identified differences in DNA methylation between nestlings reared by pairs and those reared by cooperative groups, specifically, describing the number and location of differentially methylated cytosine-phosphate-guanine (CpG) sites throughout the genome. Furthermore, we identified the genomic region and closest gene to assess whether differentially methylated sites (DMS) were present in genes associated with early nutritional and social environments. In doing so, our aim was not to quantify the number of genes related to a given function, since the impacts of methylation vary depending on where that site is found in relation to the gene, but rather to elucidate whether only nutrition-related genes were affected or whether genes linked with regulating and promoting sociality and stress regulation might also be impacted by the developmental social environment. Any such effects would have significant implications for understanding how cooperative behaviours develop within individuals over time and the fitness benefits of helping.

## 2. Material and methods

### (a) Field collections

Chestnut-crowned babblers were studied during the 2014 and 2015 breeding seasons at Fowlers Gap Research Station in far western New South Wales, Australia. Information on the study population, climate, and habitat is provided elsewhere [32,34]. Active breeding nests were identified using behavioural observations and nesting calls [38]. To determine the number of carers provisioning (and therefore visiting) young in each group, we used observations and a passive integrated transponder (PIT) tag system previously validated in this system [27,39]. More than 90% of the population is implanted subcutaneously with PIT tags, either as nestlings or as adult immigrants during their first capture. PIT tags contain a unique alphanumeric code that is recorded along with the date and time when the birds pass through a copper coil antenna connected to an LID-650 PIT tag reader (Trovan Ltd., UK). Since the antenna is attached to the entrance of their enclosed, dome-shaped nests, birds must pass through it to access the nestlings. Previous research, combining the decoder system with internal nest cameras, indicates that male carers seldom enter the nest without food (approximately 5% of entrances) and rarely fail to feed young (approximately 2.5% of the time); thus, nest visits are equivalent to provisioning events for all individuals in a group besides the breeding female, who also enters the nest to brood nestlings [40]. We can discern breeding females by both brood patches on capture and nest entrance behaviour during incubation (which is also determined using PIT tags and PIT tag readers). In addition to the breeding female, adults who entered the nest at least three times per hour across at least three days of the nestling period were considered carers (but not distinguished for breeding status or relatedness). Only nests where all adults in the breeding unit were fitted with a PIT tag and were monitored throughout nestling development were included here.

To measure chicks at hatching (see below), we removed the eggs from the nest in the latter half of the incubation period, replaced them with model eggs, and artificially incubated the eggs in a rocking incubator (Brinsea Octogon 20) at 37.5°C and 42–45% humidity until hatching. Within an hour of hatching, the chicks were weighed, their toenails were clipped for individual identification, and the chicks were returned to a nest in the field. To eliminate the effect of genetics, the nestlings were either returned to their own nest (natal) or to an unrelated nest (cross-fostered). After approximately 10 days, the chicks were ringed with a metal ring before the toenail clip grew out. At approximately 16 days (mean age = 15.8 ± 1.6 days (s.d.)), we briefly removed chicks from the nest to obtain a blood sample from a brachial vein puncture (approximately 25 µl), collected the mass, and added colour rings and a PIT tag before they were returned to the nest to fledge naturally (approximately 21 days). In total, we sampled 28 offspring from 22 broods; specifically, we used nine offspring provisioned by just their parents (five in cross-fostered, unrelated nests and four in natal nests) and 19 offspring provisioned by their parents plus additional helpers (i.e. groups; mean number of carers = 4.3 ± 1.4; seven in cross-fostered, unrelated nests and 12 in natal nests; supplementary material, table S1). Brood sizes and hatch dates among the two groups were similar (pairs: brood size 3.4 ± 1.2 (s.d.) and hatch date 1 September ± 19.9 days (s.d.); groups: brood size 3.9 ± 0.9 (s.d.) and hatch date 9 September ± 25.8 days (s.d.); supplementary material, table S1).

### (b) Molecular methods and bioinformatics

Blood samples were stored in 95% ethanol at room temperature until DNA extraction. DNA was extracted using a DNeasy (Qiagen) extraction kit according to the manufacturer's instructions. We generated converted libraries using NEBNext Enzymatic Methyl-seq (New England Biolabs), which converts unmethylated cytosines to uracils. Although bisulfite treatment has been the gold standard for analysing DNA methylation, bisulfite conversion damages DNA, whereas enzymatic conversion minimizes the damage and produces higher-quality libraries [41,42]. We performed whole-genome sequencing at a 30× depth using NovaSeq (Illumina), resulting in 18 124 698 sites across all the individuals (*n* = 28).

From the original sequencing data, we trimmed the adaptor sequences, removed low-quality sequence ends, and overlapped paired-end reads using default parameters in HTStream [43]. Paired and cleaned reads were mapped to the chestnut-crowned babbler genome [44] with an average mapping efficiency of 73% (range = 38–82%), and methylation at CpG sites was identified using Bismark [45] with the destranding option (i.e. to collapse across both strands into a single value). All sites with low coverage (less than 10×) were removed; furthermore, we removed sites with coverage in the 99.9th percentile per individual to avoid possible polymerase chain reaction bias. The proportion of DNA methylation per site was determined by dividing the number of methylated reads by the sum of methylated and non-methylated reads. Sites not present in at least 60% of both treatments (i.e. two carers and groups) were removed. CpG sites with less than 10% variation across all samples were removed to increase computational efficiency. After filtering, 1 564 780 CpG sites remained for analysis.

### (c) Data analysis of field data

The number of carers provisioning each brood was categorized as either a pair or as a cooperative group (‘three plus’ carers). To ensure that differences in the provisioning rates between pairs and groups existed, as predicted by previous work [26–28], we analysed PIT tag data from all adults at each nest (*n* = 22) after the nestlings were 10 days old. Specifically, we used a general linear mixed model (GLM) to test for the effect of the number of carers (pair versus three plus) on the number of total visits per hour to each nest; age in days of the nestling was used as a cofactor, and brood identity was used as a random effect. Additionally, we analysed the effect of carers on nestling size using GLMs in two ways. First, we tested the effect of carer number (two versus three plus) on the mass of all the nestlings (*n* = 28) on the day of sampling; brood identity was used as a random effect and cross-fostering status and age were used as cofactors to control for the fact that not all the nestlings were in natal nests or were measured at the same age, respectively. Second, we analysed the effect of carers on mass gain by subtracting the mass at hatching from the mass at the time of sampling and dividing by age in days to determine the number of grams gained per day (*n* = 26; two individuals were removed because the mass at hatching was not recorded); cross-fostering status was included as a cofactor, and brood identity was used as a random effect. GLMs were generated using lme4 [46] and lmerTest [47] in R v. 4.5.1 [48].

### (d) Data analysis of methylation

Generalized linear mixed models with a beta likelihood were used to analyse the effect of carers on the proportion of DNA methylation at each CpG site; brood identity was used as a random effect, and age in days (to account for the fact that not all individuals were measured at the same age) and cross-fostered status were used as covariates. CpG sites were considered differentially methylated (and thus associated with the number of carers present) if they passed two post hoc filters. First, only sites with a false discovery rate (FDR)-corrected *p*-value of 0.01 or less were considered significant [49]; second, to be considered biologically meaningful, sites also needed to pass an effect-size filter where the difference in the model-predicted means between the two groups was at least 25% [50]. Analyses were performed using glmmTMB [51] in R v. 4.5.1 [48]. To ensure that the models were not overinflated, we calculated the lambda for genomic inflation by comparing the Chi-square distributions of the *p*-values [52]; here, *λ* = 1.29, which is below the range of 1.33–1.72 typically seen in genome-wide methylation studies [53].

DMSs were annotated to the chestnut-crowned babbler reference genome (NCBI accession: GCA_013400735.1). Specifically, the CpG site coordinates were converted to Granges objects in R and overlapped with the annotation using the GenomicRanges package [54]. In addition, we defined the genomic location of each CpG site as follows: the transcription start site (TSS; 300 bp upstream to 50 bp downstream of the annotated gene start site), the promoter region (2000 bp upstream to 200 bp downstream of the annotated start site), the gene body, and the 5′ or 3′ untranslated region (5′UTR and 3′UTR, respectively; 10 000 bp in either direction of the gene body). To determine whether any of the DMSs were over-represented in or near genes involved in biological pathways, we used two approaches. First, because the presence of multiple DMSs in close proximity to one another may be indicative of increased functional regulation of genes [55], we also identified differentially methylated regions (DMRs), defined as three or more carer-linked CpG sites within a 2 kb window using the dmrff package [56] in R [48]; although smaller windows are often used with reduced representation data, larger windows (i.e. between 500 and 2000 bp) are recommended for genome-wide studies [57,58]. Second, sites found in the TSS, promoter, gene body, and UTRs (i.e. CpG sites located in intergenic DNA were removed from functional interpretation) were used in a gene ontology (GO) analysis using g:Profiler with humans as a reference [59]. GO terms with an FDR less than 0.05 were considered significantly over-represented.

## 3. Results

**Figure 1.**
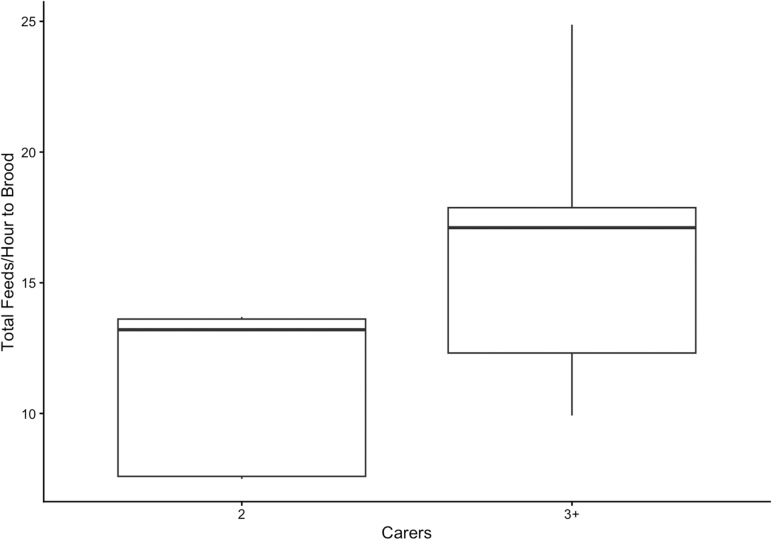
Differences in feeds per hour between nestlings raised by a pair and those raised by a group (three plus (3+)). For each group, the box represents the full interquartile range, with the middle line showing the data median.

**Figure 2.**
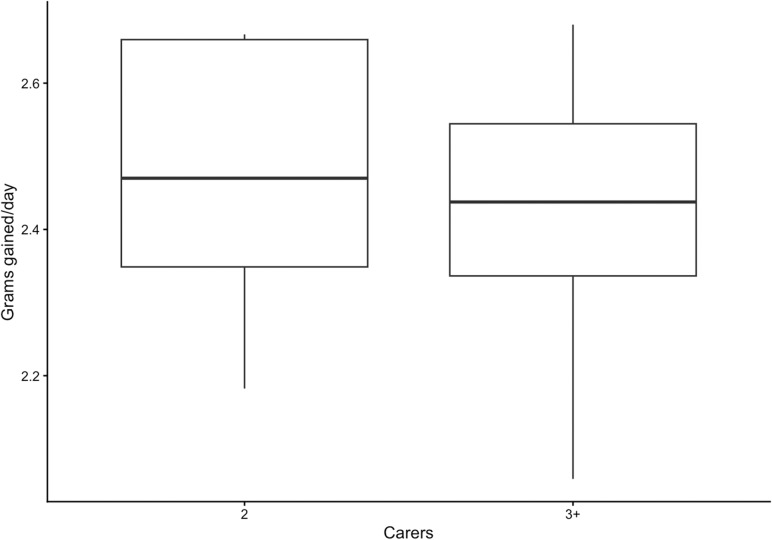
Differences in mass gained per day (g) in nestlings raised by a pair and those raised by a group (three plus (3+)) throughout the nestling developmental period. For each group, the box represents the full interquartile range, with the middle line showing the data median. Like mass gain (here), size at the time of the last sampling did not differ between the nestlings raised by pairs and those raised by groups (data not shown).

As expected, differences existed in the social and nutritional environments of the nestlings raised by pairs and those raised by groups. Nestlings raised by groups were visited and provisioned at the nest more often than nestlings raised by pairs (*t* = 2.18, *p* = 0.037; estimate = 4.5 ± 2.1; figure 1). Despite this, neither metric of nestling size differed between the two groups: neither the mass of the last sample (*t*_1,24_ = 0.29, *p* = 0.77) nor the grams gained per day (*t*_1,16.9_ = −0.28, *p* = 0.79; figure 2) differed between the nestlings raised by pairs or groups. Cross-fostering status did not predict any of the results (*p* > 0.6), but age, unsurprisingly, did predict the mass at last sampling (*p* = 0.012; supplementary material).

**Figure 3.**
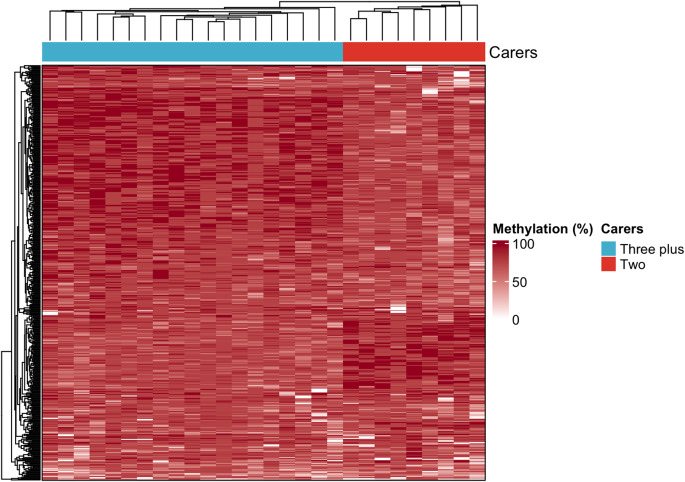
Hierarchical clustering of differentially methylated sites in chestnut-crowned babbler nestlings. Each row represents one of the top 500 sites that were significantly different between individuals with two carers (red) or groups (three plus carers; blue). The colour scale represents the percentage of DNA methylation, with higher DNA methylation indicated by a darker shade of red.

Among the 1 564 780 CpG sites tested in the analysis, a total of 1057 were differentially methylated between nestlings raised by just parents and those raised by groups (figure 3). Most sites (867; 82%) were hypermethylated in nestlings raised by groups compared with those raised by pairs. Although many of the DMSs were in intergenic regions (46%), 570 were associated with annotated genes; specifically, eight were found in the TSS, 34 in the promoter, 307 in the gene body, and 221 in the UTR, either upstream or downstream of the gene (supplementary material, figure S1 and table S2). Similarly to the total number of DMSs, the majority of the DMSs associated with genes (85%) were hypermethylated in chicks raised by groups compared with those raised by only two carers; this was true across all gene-associated locations (i.e. 88% of sites located in the TSS, 85% of those in the promoter, 85% of those in the gene body, and 85% of those in the UTR had higher levels of DNA methylation in nestlings raised by groups). No DMRs (regions with three or more DMSs within a 2 kb window) associated with the number of carers were identified.

**Figure 4.**
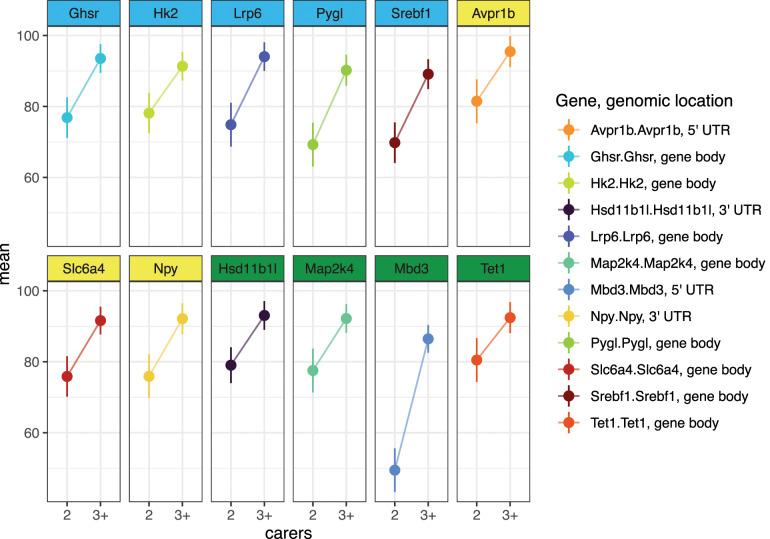
Differentially methylated sites in genes important for metabolism and growth (blue headings), for the regulation and promotion of sociality (yellow headings), and for the regulation of the stress response (green headings). DNA methylation is given as the average ± standard error in nestlings reared by two adults versus groups (three plus (3+)). Point colours represent the genomic location, given as the closest gene, the regulatory body (i.e. gene body or 3′ untranslated region (UTR)), and the number of bases away from the transcriptional start site. DNA methylation was generally higher in nestlings reared by groups, but not at every site.

The 570 DMSs in regulatory locations mapped to a total of 487 genes, generally with only a single DMS per gene. Among the 28 genes with multiple DMSs, 27 had two associated DMSs and one had three. DMSs associated with the same gene were largely methylated in the same direction (i.e. were all more methylated in nestlings reared by groups or were all less methylated in nestlings reared by groups), but four genes contained sites that were methylated in opposite directions. GO term analysis revealed 18 terms that were significantly over-represented, with an FDR of 0.05, including those related to protein binding, development, cell communication and signalling, and response to internal and external stimuli (table 1), indicating that differences in carer numbers may be particularly important for these pathways in developing nestlings. In addition, several DMSs were found to be associated with genes important for growth and metabolism (e.g. *GHSR, HK2, LRP6, PYGL* and *SREBF1*), genes associated with regulating or promoting sociality (e.g. *AVPR1b, SLC4A4* and *NPY*), and genes associated with the stress response or communicating environmental stressors to the phenotype (e.g. *HSD11b1l, MAP2K4, MBD3* and *TET1*; figure 4; supplementary material, table S2).

**Table 1.** Over-represented GO terms (i.e. those with an FDR-adjusted *p*-value < 0.05) in the DMSs in regulatory regions (i.e. transcriptional start sites, promoters, gene bodies, and untranslated regions) influenced by carer number experienced by nestlings before fledging.

GO term ID	FDR adjusted *p*	GO term name	description
**molecular function GO terms**			
GO:0005515	1.41E-04	protein binding	proteins attaching to other proteins to enable cell communication and regulation
GO:0004725	1.5E-4	protein tyrosine phosphatase activity	important for regulating cell growth and proliferation and metabolic homeostasis
GO:0005244	3.8E-2	voltage-gated monoatomic ion channel activity	important for action potentials in neurons and muscle cells
**biological process GO terms**			
GO:0048731	1.1E-9	system development	growing and shaping tissues and organs during development
GO:0034330	3.02E-6	cell junction organization	important for cell-to-cell and cell-to-environment communication
GO:0007155	1.69E-4	cell adhesion	important for cell-to-cell communication and growth
GO:0023051	1.34E-3	regulation of signalling	determines how cells respond to internal and external cues
GO:0010646	1.49E-3	regulation of cell communication	involved in cell-to-cell and cell-to-environment communication
GO:0098976	1.89E-3	excitatory chemical synaptic transmission	synaptic transmission resulting in an excitatory postsynaptic potential
GO:2000794	5.45E-3	regulation of epithelial cell proliferation involved in lung morphogenesis	promoting cell growth in lung epithelial cells
GO:0051716	0.02	cellular response to stimulus	processes that respond to stimuli that induce a change in state or activity of a cell
GO:0007166	0.03	cell surface receptor signalling pathway	induced by binding of extracellular ligands to receptors on the cell surface
**cellular component GO terms**			
GO:0030054	1.47E-6	cell junction	connections between cells important for cell-to-cell communication
GO:0045121	1.04E-4	membrane raft	act as a platform to organize signalling molecules and receptors
GO:0005737	3.42E-4	cytoplasm	important for functions occurring in the cytoplasm, including energy production and translation
GO:0098588	9.74E-3	bounding membrane of organelle	membrane bound proteins in organelles like mitochondria
GO:1990351	0.01	transporter complex	facilitates molecular transport among cells, promoting cell-to-cell communication
GO:0043005	0.05	neuron projection	induces neural plasticity and connections

## 4. Discussion

We present, to our knowledge, one of the first studies to examine the effect of having additional carers on genome-wide levels of DNA methylation in a cooperatively breeding vertebrate. First, we found that nestlings reared by groups (i.e. parents plus additional carers) were visited more often than those reared by pairs, owing to the greater number of provisioners present to do so. Given that the vast majority of visits reflect provisioning events [40] and that visitation rates are positively associated with the biomass of food delivered per hour [27], nestlings reared by groups probably receive more food than those reared by pairs. Although there was no effect on nestling mass, previous work has shown that nestlings reared in groups are less likely to die from starvation [25]. Thus, it is likely that nestlings reared by groups are able to allocate energy to other predictors of survival, rather than mass per se, such as immune function [60] or feather growth [61], which may lead to earlier fledging. Second, nestlings reared in groups had higher overall DNA methylation; furthermore, we identified differential DNA methylation at 570 CpG sites related to annotated genes, including some important for metabolism and growth, the regulation and promotion of sociality, and the ability to cope with environmental stressors. These results suggest that information about the nutritional and social environment experienced during early development influences molecular marks on the genome, with potential implications for future phenotypes.

The specific effect of DNA methylation is often dependent on the genomic location in which it is found and can even be specific to each CpG. For instance, DNA methylation of a CpG site in the promoter region of a gene, particularly the transcriptional start site, is generally considered to be inhibitory of transcription factor binding [62–64]; however, some transcription factors preferentially bind to methylated binding sites [65] or even require DNA methylation to bind [66,67]. Furthermore, in the gene body, DNA methylation has several functions, including the ability to control alternative promoters [68], determine alternative splicing [69], or suppress transposable element expression [64]. Finally, methylation in either the 3′ or 5′ UTR can also regulate gene expression, although the mechanisms are less well understood [70]. Although assessing the full phenotypic impacts of the DMSs identified here is difficult, the key message is that differential nutritional and social environments during development are related to predictable differences in DNA methylation, including at sites relevant to genes related not only to growth and metabolism but also those responsible for regulating and promoting sociality, coping with stressors, and cell communication. Taken together, these findings suggest that helpers in cooperative breeding systems have a greater effect on offspring outcomes than just a straightforward nutritional benefit. Using both nutritional and social information gained during development, nest-bound chicks are likely to gain information about their future environment, including resource availability, social structure, and competition. Thus, understanding how developmental environments shape molecular markers that may drive future phenotypic consequences is important for advancing our understanding of how cooperative care may influence offspring beyond the simple short-term benefits of added nutrition.

In this study, we assessed DNA methylation in red blood cells. Some of the genes identified here with DMSs are neurotransmitters whose mode of action and expression are focused in the brain; thus, the epigenetic patterns derived using blood samples might not be representative of those patterns found elsewhere in the body [71,72]. However, significant relationships between DNA methylation in blood and the brain have been identified in wild birds [73]. Furthermore, the use of non-lethal samples offers a mechanism to enhance eco-epigenetic studies by allowing repeated sampling over time, particularly during important developmental windows when genomes might be expected to be more reactive to changes in the environment. Finally, using less invasive sampling methods allows the impact of developmental DNA methylation on later phenotypic trajectories to be assessed. Importantly, meaningful relationships between DNA methylation in blood samples and both environmental and phenotypic variation have been identified in other species (primarily humans, where blood samples are generally the only option). For instance, increased levels of parental care in humans are related to decreased DNA methylation of *OXTR* and *BDNF* extracted from blood samples [22]. A recent study in house sparrows (*Passer domesticus*) revealed differentially methylated sites in the promoters of stress-regulating genes (e.g. *NR3C1, NR3C2, HSD11B1)* in the blood related to early life and intergenerational stress [74]. These studies suggest that using blood samples to measure DNA methylation patterns is a reliable means of identifying salient relationships with environmental variability, even when genes primarily expressed in the brain are used; however, the results need to be interpreted with this in mind, and future research might consider validating these findings across tissues.

Additional nutritional resources are often considered to be the main benefit of carers in cooperatively breeding species and have been demonstrated to have significant short-term effects on the growth and survival of offspring [4,5]. In other, noncooperative species, differential resource availability directly affects DNA methylation [50,75]. For example, in yellow baboons (*Papio cynocephalus*), variation in access to food induces genome-wide differential methylation patterns that persist even after individuals disperse into areas with more food [75]. Here, confirming previous work [29], we show that nestlings reared by groups receive more visits to the nest; given that visits are equivalent to provisioning [40], we also know that nestlings reared by groups receive more food compared to those raised with pairs. In line with that, we also found evidence of differential DNA methylation in the genome in areas related to differences in the nutritional environment. First, the GO term analysis revealed multiple areas of over-representation, including at least two directly related to development and growth (protein tyrosine phosphatase activity and system development). Second, multiple DMSs were identified in regions associated with growth and metabolic regulation, including those that regulate hunger signals (e.g. *GHSR*) and those that regulate glucose metabolism (e.g. *HK2, LRP6,* and *PYGL*) and fatty acid synthesis (e.g. *SREBF1*). However, neither final mass nor mass gain differed between the two groups, suggesting that, unlike in other species [2,4], additional helping in chestnut-crowned babblers may not influence nestling mass. This is not necessarily contrary to the differential methylation results we reported for genes related to metabolism and growth. Rather, the fact that multiple sites throughout the regulatory regions of genes related to metabolism and growth were differentially methylated suggests that nestlings reared by groups probably respond to metabolic and growth demands differently than those reared by just their parents; just as there are multiple ways to skin a cat, there are multiple ways to achieve a competitive size at fledging, and these are probably regulated through epigenetic mechanisms. Although nestlings reared in larger groups are brought more food throughout the nestling period, physiological shifts in metabolic performance might increase the efficiency of nestlings reared in pairs [76]. It is unclear whether physiological shifts in metabolic function are detrimental to nestlings in either the short or long term, as has been demonstrated in other species [77]. Additionally, we do not know how consistent differences in DNA methylation are in chestnut-crowned babbler nestlings across life stages and whether early-life determination of life-time metabolic and growth strategies via DNA methylation is adaptive (but see [75]). Taken together, these correlative results highlight the need to better understand the relationship between methylation at particular sites and specific changes in phenotypic function.

In addition to added nutrition, carers provide information regarding the expected social environment postfledging. Unlike the nutritional impacts of additional carers, which are largely linear [26,27], the social influence of an increasing number of helpers is largely unknown; however, we hypothesize that the increases in social learning, memory, and prosocial behaviour necessary to cope with larger groups are not linear. In particular, the transition from parental care alone to the presence of at least one helper may require a qualitatively different phenotype, whereas the addition of subsequent helpers may represent a comparatively minor extension of an already established social framework. Here, we found DMSs in regulatory areas associated with genes that are expected to be important for regulating and promoting sociality and coping with stressors. For instance, one of the most studied neuropeptides that regulates sociality among model organisms is arginine vasopressin, which, in conjunction with its receptors, plays a pivotal role in the regulation of complex social behaviour, regulating variation in parental care, pair bonding, social memory, and social aggression [78–80]. In other species, reduced expression of *AVPRB* in the brain leads to reductions in competition, territoriality, and recognition abilities [81,82]; furthermore, studies have suggested a role for *AVPRB* in the regulation of the HPA pathway [83]. Further evidence of the effect of the developmental social environment on social behaviour in nestling chestnut-crowned babblers comes from the differential methylation of a CpG site associated with the serotonin receptor (*SLC6A4*) and neuropeptide Y (*NPY*), both of which regulate social behaviour by increasing prosocial and cooperative behaviour and reducing aggression in other species [84,85]. Additionally, we identified a DMS in the regulatory region of *HSD11b1l*, an enzyme responsible for regulating the availability of physiologically relevant glucocorticoids [86], which are important in directing an individual's physiological and behavioural response to environmental stressors [87].

Finally, for nestlings to respond appropriately to any environment, including the social rearing environment, they must be able to receive and respond to cues from their environment, and then cells must be able to communicate those cues to other cells to generate an organismal response. Interestingly, of the 18 GO terms that were over-represented in our data, at least 13 were related to the cellular response to external stimuli and cell-to-cell communication. Furthermore, DMSs were detected throughout the regulatory regions of genes important for epigenetic regulation, such as *MDB3* and *TET1*. Epigenetic regulators perceive and respond to changes in the environment to promote molecular, physiological, and behavioural changes to cope with perturbations as they arise, thus acting as a mechanism to respond to changes in the environment [88]. *MAP2K4* is also critical for the response to external stressors and the regulation of stress response pathways such as oxidative stress, heat shock, and disease. Together, these findings indicate that differences in the social environment lead to differences in the methylation of genes involved in pathways that underlie environmental signal detection, signal transduction and cell-to-cell communication, all of which underlie differences in phenotypic plasticity, providing a potential mechanistic link between the early-life social environment and organismal response.

Notably, the patterns we describe here are the result of a correlative study. The implications of the conclusions we draw are somewhat dependent on three things. First, we assume that the differences we observe are related to the developmental social environment and not a different environmental trait. Although it is possible that other environmental variables drive the differences we observed, most of a nestling chestnut-crowned babbler's environment is driven by social group size from food delivery [26–28] to the consistency of brooding temperature [89]. Second, we assume that the differences we report are a result of the social environment during the nestling period; however, an alternative possibility is that they arise from prehatching. Even if this were true, it is likely that any such effect would also be socially mediated. For example, in the cooperative breeding of superb fairy wrens (*Malurus cyaneus*), the nutritional content of mothers varies as a function of helper number [90]. In addition, in chestnut-crowned babblers, mothers incubate more in groups than in pairs, leading to more constant egg temperatures during development [89]. However, by using a cross-fostering design and keeping the eggs in consistent common-garden incubation environments (i.e. incubators), any effect of the prehatching period should be minimal. Third, the impact of the results we report here might be dependent on their persistence through time. Although the persistence of differential DNA methylation patterns into adulthood in chestnut-crowned babblers is currently unknown, it is improbable that all the DNA methylation differences we observed would remain established throughout life; equally, however, it is also improbable that all the differences would erase on fledging. Theoretically, DNA methylation should occur when the rearing environment forecasts future environments [91–93]. Indeed, individuals reared by larger groups, by definition, will always have more kin than those raised by pairs, which has implications for nepotistic benefits [36] and opportunities for helping versus breeding in the future [30].

## 5. Conclusion

The results of this study point to a mechanism by which carers directly influence offspring in cooperative breeders, potentially to prepare offspring for the future environment which they will ultimately experience postfledging. Given the accumulating evidence that conditions experienced during development are crucial in inducing stable phenotypes via epigenetic modifications [20,94], it might be expected that the differences in DNA methylation observed here lead to predictable phenotypic changes. Furthermore, if DNA methylation in response to differences in the social environment is priming individuals to best suit future environments, we might expect these changes (both molecular and phenotypic) to be permanent. Two key aims for future studies should be to determine the stability of such epigenetic signals established early in life and their lasting phenotypic effect, to determine whether the patterns we detected here are causal drivers of variation in carers during development or are simply correlated with these environments. Although the manipulations required to determine a causal effect are difficult in wild animals, future work may consider adult removal to manipulate carer number. A simpler alternative might be to manipulate carer : offspring ratios through brood size manipulations, which are known to induce effects on DNA methylation [95–97], although previous research in this system suggests that the effect of an increased brood is not necessarily proportional to the effect of reduced carers provisioning at the nest [28].

At the individual level, the developmental social environment affects how individuals interact with their environments and dictates variations in behaviours ranging from exploration to anti-predatory behaviours, to foraging success; in cooperatively breeding species, these impacts of early-life social environments might even dictate an individual's likelihood of cooperating later in life [98]. The results here provide a first step in understanding the molecular mechanisms through which early-life social environments influence the molecular markers that are likely to play a role in determining downstream phenotypes. As climates rapidly change, populations facing environmental stressors, such as those experienced in harsh, arid environments, may require a high degree of flexibility to cope and survive. Such levels of flexibility may be facilitated in social animals via communication shared through developmental social environments. The developmental social environment is likely to be a key yet largely overlooked factor that shapes rather than limits the potential for phenotypic plasticity through DNA methylation.

## Supplementary Material



## Data accessibility

All the code used for the analyses is available at https://github.com/sca987/Birds_WG_meth. Data are available through NCBI GEO accession number GSE310508.

## Theme

One contribution of 17 to a theme issue ‘Ecological epigenetics at the intersection of behaviour and life history variation in non-model animals’.
