# Supplementary material for: Cooperative care influences genome-wide levels of DNA methylation in nestling chestnut-crowned babblers
Source: Philos Trans R Soc Lond B Biol Sci. 2026 Jul 23;381(1955):rstb20250014. doi: 10.1098/rstb.2025.0014 (PMC13479371; doi:10.1098/rstb.2025.0014)
Supplement: Supplementary file 1 [file rstb20250014.zip › rstb20250014/rstb20250014_si_001.docx]

**Supplemental Results**

*Co-factor relationships with field-collected data*

Age did not predict provisioning rate of chicks between pairs and groups (F_1, 139_ = 0.78, p = 0.44).

Cross-fostering status did not influence mass at sampling (t_1,24_ = -0.24, p = 0.8), nor grams gained per day (t_1,25_ = -0.55, p = 0.6); however, unsurprisingly, age did determine mass at sampling (F_1,24_ = 2.71, p= 0.012) with older nestling being significantly bigger.

Supplemental Table 1. Information about each of the individuals included in the analysis of the effect of carer number on methylation throughout the genome of chestnut-crowned babbler nestlings.

| sample | carer category | number of carers | cross-fostered | age at last sample | mass at last sample | brood size |
| --- | --- | --- | --- | --- | --- | --- |
| 1 | 3+ | 3 | n | 18 | 45 | 5 |
| 2 | 3+ | 3 | n | 19 | 41 | 3 |
| 3 | 2 | 2 | y | 19 | 42 | 3 |
| 4 | 2 | 2 | y | 16 | 38 | 4 |
| 5 | 3+ | 3 | n | 20 | 44.5 | 4 |
| 6 | 3+ | 3 | y | 14 | 38 | 3 |
| 7 | 2 | 2 | n | 15 | 40 | 6 |
| 8 | 3+ | 4 | y | 17 | 47.5 | 5 |
| 9 | 2 | 2 | y | 17 | 40 | 3 |
| 10 | 3+ | 5 | n | 17 | 42.5 | 4 |
| 11 | 3+ | 4 | n | 14 | 38.5 | 4 |
| 12 | 3+ | 3 | y | 17 | 38 | 4 |
| 13 | 3+ | 8 | n | 14 | 41.5 | 5 |
| 14 | 3+ | 3 | y | 16 | 43 | 3 |
| 15 | 3+ | 3 | n | 16 | 45.5 | 3 |
| 16 | 2 | 2 | n | 14 | 40.5 | 4 |
| 17 | 2 | 2 | n | 16 | 41 | 4 |
| 18 | 3+ | 4 | n | 15 | 38 | 2 |
| 19 | 3+ | 6 | n | 16 | 39.5 | 4 |
| 20 | 3+ | 6 | y | 16 | 42 | 4 |
| 21 | 3+ | 3 | y | 15 | 43 | 3 |
| 22 | 2 | 2 | n | 15 | 43 | 3 |
| 23 | 3+ | 5 | n | 14 | 35.5 | 3 |
| 24 | 2 | 2 | y | 14 | 40.5 | 2 |
| 25 | 2 | 2 | y | 15 | 39.5 | 2 |
| 26 | 3+ | 5 | n | 15 | 39 | 5 |
| 27 | 3+ | 5 | y | 14 | 36 | 5 |
| 28 | 3+ | 5 | n | 15 | 42 | 5 |

Supplemental Figure 1. Percent differentially methylated sites associated with carer number (controlling for age and cross-fostering status) before fledging were distributed throughout the genome in both regulatory (including transcriptional start site, promoter, gene body, and untranslated regions) and nonregulatory regions (i.e. intergenic).

Supplemental Table 2. List of significant differentially methylated sites associated with genes with location in the genome, distance from transcriptional start site (TSS), ID of closest gene, estimate, model-predicted means of nestlings reared by a pair and those reared by a group (3+), and the FDR-adjusted p-value.

| Site ID | Genomic location | distance to TSS | Gene ID | Estimate | Model-predicted mean (pair) | Model-predicted mean (3+) | FDR adjusted p-value |
| --- | --- | --- | --- | --- | --- | --- | --- |
| VYZT01021241.1_46551 | TSS | -8 | Agxt | 2.52 | 0.69 | 3.22 | 3.98E-17 |
| VYZT01017305.1_9781 | TSS | -286 | Cpxm1 | 1.92 | 0.59 | 2.52 | 9.62E-03 |
| VYZT01013284.1_182016 | TSS | -23 | Cux1 | 2.56 | 0.74 | 3.30 | 1.93E-04 |
| VYZT01059399.1_36406 | TSS | -163 | Krt17 | 2.59 | 0.05 | 2.64 | 1.92E-21 |
| VYZT01000040.1_108735 | TSS | -186 | Nfkbiz | -2.02 | 2.83 | 0.81 | 9.58E-03 |
| VYZT01013284.1_471000 | TSS | -45 | Por | 1.82 | -0.86 | 0.96 | 1.33E-03 |
| VYZT01014638.1_249109 | TSS | -98 | Rasal2 | 2.44 | 0.47 | 2.91 | 1.67E-03 |
| VYZT01059376.1_104120 | TSS | -10 | Vezf1 | 1.89 | 0.40 | 2.29 | 5.48E-05 |
| VYZT01033829.1_113322 | promoter | -582 | Ambp | 1.79 | 0.70 | 2.49 | 7.82E-03 |
| VYZT01049427.1_226779 | promoter | -1762 | Bsn | 1.17 | 0.05 | 1.22 | 0.00E+00 |
| VYZT01048653.1_152879 | promoter | -652 | Cacna2d2 | 2.76 | -2.04 | 0.72 | 8.32E-03 |
| VYZT01044762.1_193059 | promoter | -943 | Ccdc63 | 1.57 | 0.52 | 2.09 | 6.18E-31 |
| VYZT01007618.1_30899 | promoter | -1551 | Cmlkr1 | -2.23 | 2.91 | 0.68 | 1.12E-03 |
| VYZT01031655.1_9587 | promoter | -577 | Crispld2 | -1.29 | 1.64 | 0.35 | 1.02E-26 |
| VYZT01011807.1_14332 | promoter | -1424 | Eppk1 | 4.35 | 0.14 | 4.49 | 0.00E+00 |
| VYZT01062667.1_21659 | promoter | -1289 | Fam49a | 2.65 | -0.63 | 2.02 | 1.62E-25 |
| VYZT01025844.1_91838 | promoter | -1046 | Fgfr3 | 2.10 | 0.67 | 2.77 | 9.50E-03 |
| VYZT01000035.1_50056 | promoter | -1554 | Fmnl1 | 2.74 | 0.76 | 3.50 | 6.35E-07 |
| VYZT01052924.1_90222 | promoter | -982 | Fmo5 | 1.64 | -0.64 | 0.99 | 6.49E-08 |
| VYZT01062670.1_98589 | promoter | -920 | Galm | 1.08 | -0.36 | 0.73 | 6.28E-04 |
| VYZT01062697.1_89912 | promoter | -1530 | Gjb1 | -1.34 | 1.68 | 0.34 | 9.28E-05 |
| VYZT01028158.1_31785 | promoter | -884 | H2afz | 2.13 | 0.81 | 2.94 | 3.74E-03 |
| VYZT01053458.1_17949 | promoter | -1672 | Hes5 | 2.68 | 0.77 | 3.45 | 2.53E-04 |
| VYZT01015816.1_552336 | promoter | 74 | Kcnj4 | 2.07 | 0.82 | 2.89 | 9.74E-03 |
| VYZT01020443.1_24412 | promoter | 101 | Lingo3 | 1.72 | 0.67 | 2.39 | 4.97E-03 |
| VYZT01044770.1_115350 | promoter | -1058 | Muc1 | 1.98 | -0.40 | 1.58 | 6.72E-25 |
| VYZT01019215.1_259011 | promoter | 1029 | Nop16 | -2.11 | 2.89 | 0.78 | 9.40E-03 |
| VYZT01041606.1_18199 | promoter | 85 | Perp | 1.19 | -0.14 | 1.05 | 4.19E-09 |
| VYZT01012437.1_82907 | promoter | -1759 | Pitx3 | 2.18 | -1.10 | 1.09 | 5.37E-04 |
| VYZT01020518.1_65138 | promoter | -1846 | Ptchd1 | 1.42 | 0.33 | 1.75 | 6.95E-03 |
| VYZT01006055.1_5897 | promoter | -900 | Ror1 | 1.80 | 0.23 | 2.04 | 3.48E-07 |
| VYZT01004136.1_942 | promoter | -885 | Ryk | 1.10 | -0.60 | 0.50 | 0.00E+00 |
| VYZT01007905.1_98223 | promoter | -365 | St3gal2 | 2.07 | -1.11 | 0.95 | 3.84E-03 |
| VYZT01058892.1_4891 | promoter | 72 | Tbx4 | 1.92 | 0.70 | 2.62 | 7.26E-03 |
| VYZT01056678.1_69355 | promoter | 612 | Tmub1 | 1.81 | 0.59 | 2.40 | 4.51E-03 |
| VYZT01021163.1_96034 | promoter | -1952 | Tns4 | 2.01 | 0.43 | 2.44 | 5.73E-03 |
| VYZT01028644.1_49956 | promoter | 54 | Usp31 | -1.87 | 2.56 | 0.69 | 7.39E-03 |
| VYZT01017291.1_367272 | promoter | -686 | Wdr90 | 2.10 | 0.24 | 2.34 | 3.20E-03 |
| VYZT01044777.1_2083 | promoter | -1246 | Znf397 | 2.05 | 0.69 | 2.74 | 7.32E-03 |
| VYZT01048226.1_56169 | gene body | 244 | Abcb9 | 2.85 | 0.54 | 3.39 | 1.41E-05 |
| VYZT01036594.1_246879 | gene body | 24024 | Abcc1 | 2.15 | 0.67 | 2.82 | 2.36E-03 |
| VYZT01024187.1_39662 | gene body | 2040 | Abcc8 | 2.24 | 0.76 | 3.00 | 3.42E-03 |
| VYZT01008922.1_159554 | gene body | 12930 | Abcc9 | -1.13 | 1.03 | -0.09 | 9.77E-03 |
| VYZT01007738.1_50864 | gene body | -8732 | Abtb1 | 2.04 | 0.61 | 2.65 | 4.42E-03 |
| VYZT01021292.1_202078 | gene body | 3146 | Acbd3 | 2.15 | 0.76 | 2.91 | 1.09E-03 |
| VYZT01035974.1_25819 | gene body | 4035 | Acsf3 | 2.93 | -1.44 | 1.49 | 9.13E-03 |
| VYZT01035974.1_42899 | gene body | 21115 | Acsf3 | 2.06 | 0.22 | 2.29 | 3.36E-03 |
| VYZT01123305.1_729 | gene body | 322 | Acvr1c | -2.15 | 2.89 | 0.74 | 4.66E-03 |
| VYZT01036593.1_27811 | gene body | 1495 | Adam33 | 2.13 | 0.80 | 2.92 | 4.15E-03 |
| VYZT01063914.1_44800 | gene body | 79583 | Agap1 | 2.20 | 0.62 | 2.82 | 8.76E-04 |
| VYZT01063914.1_39603 | gene body | 84780 | Agap1 | 1.97 | 0.62 | 2.59 | 5.87E-03 |
| VYZT01025100.1_44920 | gene body | 29493 | Agap3 | 3.36 | 0.18 | 3.54 | 3.97E-10 |
| VYZT01065389.1_35654 | gene body | 205 | Aicda | -1.90 | 2.62 | 0.73 | 8.38E-03 |
| VYZT01033829.1_185731 | gene body | 1085 | Ajm1 | 2.41 | 0.88 | 3.28 | 4.99E-05 |
| VYZT01056725.1_442305 | gene body | 17225 | Akap13 | 2.27 | 0.69 | 2.96 | 7.35E-03 |
| VYZT01028662.1_147235 | gene body | 4986 | Aldh3a2 | 2.50 | 0.88 | 3.38 | 1.27E-05 |
| VYZT01050659.1_61777 | gene body | 36738 | Ankrd33b | -1.95 | 2.67 | 0.72 | 6.12E-03 |
| VYZT01032166.1_100382 | gene body | 7944 | Anks1a | 1.35 | -0.37 | 0.98 | 4.94E-48 |
| VYZT01061351.1_8150 | gene body | 410 | Arc | 2.29 | 0.05 | 2.34 | 8.73E-22 |
| VYZT01000608.1_126334 | gene body | 5417 | Arhgap19 | 1.96 | 0.74 | 2.70 | 5.30E-04 |
| VYZT01032661.1_43297 | gene body | 15394 | Arhgap21 | -2.20 | 3.04 | 0.84 | 4.19E-03 |
| VYZT01022039.1_4852 | gene body | 1422 | Arhgap39 | -7.14 | 7.62 | 0.47 | 1.10E-14 |
| VYZT01025069.1_151457 | gene body | 14977 | Arid3a | 2.18 | 0.73 | 2.91 | 2.09E-03 |
| VYZT01035271.1_15157 | gene body | 2195 | Arid5b | 1.87 | 0.64 | 2.50 | 2.43E-03 |
| VYZT01004115.1_186918 | gene body | 21134 | Arsj | 2.77 | 0.12 | 2.89 | 5.88E-05 |
| VYZT01021331.1_29258 | gene body | 21957 | Ash1l | -2.03 | 2.81 | 0.78 | 3.63E-04 |
| VYZT01044832.1_227511 | gene body | 2346 | Asphd2 | 2.15 | 0.77 | 2.92 | 6.39E-03 |
| VYZT01022492.1_29177 | gene body | 16059 | Astn1 | -2.04 | 2.78 | 0.74 | 1.81E-03 |
| VYZT01050670.1_189326 | gene body | 34029 | Astn2 | 2.14 | -0.46 | 1.67 | 7.78E-05 |
| VYZT01029259.1_4016 | gene body | 3551 | Atp13a2 | 1.47 | -0.04 | 1.43 | 0.00E+00 |
| VYZT01016580.1_65309 | gene body | 36144 | Atp2a2 | 2.01 | 0.52 | 2.54 | 4.35E-03 |
| VYZT01007598.1_66197 | gene body | 28400 | Atp9a | 1.55 | 0.52 | 2.07 | 7.74E-03 |
| VYZT01032110.1_9073 | gene body | 89121 | B3gntl1 | 2.04 | 0.26 | 2.30 | 7.45E-03 |
| VYZT01032110.1_45458 | gene body | 52736 | B3gntl1 | 2.10 | 0.78 | 2.89 | 9.67E-03 |
| VYZT01040209.1_113646 | gene body | 749 | B4galt7 | 1.86 | 0.76 | 2.62 | 4.03E-03 |
| VYZT01029813.1_255387 | gene body | 2769 | Bcar1 | 1.93 | 0.07 | 2.00 | 8.82E-03 |
| VYZT01035958.1_415797 | gene body | 17864 | Bcl2l13 | 2.13 | 0.85 | 2.97 | 5.26E-03 |
| VYZT01029258.1_16394 | gene body | 2279 | Bcs1l | 2.92 | 0.83 | 3.75 | 7.75E-06 |
| VYZT01016176.1_157478 | gene body | 7902 | Bnip3l | 1.99 | 0.80 | 2.79 | 5.36E-03 |
| VYZT01009752.1_128983 | gene body | 30289 | Btbd6 | 2.20 | 0.72 | 2.92 | 5.22E-03 |
| VYZT01000749.1_14660 | gene body | 1578 | Btn1a1 | -1.60 | 2.12 | 0.52 | 7.79E-158 |
| VYZT01041517.1_16779 | gene body | 8189 | Cacnb4 | 1.88 | 0.73 | 2.62 | 8.82E-03 |
| VYZT01003450.1_252891 | gene body | 10034 | Capn8 | 1.94 | 0.78 | 2.72 | 6.46E-03 |
| VYZT01026412.1_196220 | gene body | 8726 | Card11 | 5.71 | -2.65 | 3.06 | 1.32E-07 |
| VYZT01062722.1_196023 | gene body | 2789 | Ccdc157 | -2.63 | 2.17 | -0.46 | 7.86E-36 |
| VYZT01035950.1_12664 | gene body | -7801 | Ccdc81 | 2.58 | 0.52 | 3.10 | 4.25E-03 |
| VYZT01026490.1_128277 | gene body | 43107 | Ccdc85a | 1.36 | -0.20 | 1.16 | 0.00E+00 |
| VYZT01037715.1_146914 | gene body | -4955 | Cd48 | 1.39 | -0.32 | 1.07 | 0.00E+00 |
| VYZT01049385.1_40023 | gene body | 170889 | Cdh13 | 2.22 | 0.63 | 2.86 | 7.18E-04 |
| VYZT01056122.1_40104 | gene body | 6030 | Clint1 | 2.14 | 0.85 | 2.99 | 2.33E-03 |
| VYZT01028128.1_18651 | gene body | 1322 | Clp1 | 1.76 | 0.53 | 2.29 | 7.86E-03 |
| VYZT01063217.1_73432 | gene body | 1438 | Cog1 | 1.89 | 0.70 | 2.59 | 2.99E-03 |
| VYZT01001521.1_73529 | gene body | 4866 | Coq3 | 1.74 | -0.28 | 1.47 | 5.21E-03 |
| VYZT01058449.1_52199 | gene body | -31712 | Cox7a2 | 1.86 | 0.40 | 2.26 | 6.46E-03 |
| VYZT01015811.1_279064 | gene body | -2138 | Cpne9 | 2.23 | 0.44 | 2.67 | 6.33E-03 |
| VYZT01038275.1_12205 | gene body | -4624 | Ctsh | 2.72 | 0.47 | 3.19 | 1.44E-29 |
| VYZT01024984.1_141519 | gene body | 252 | Cxcr1 | -1.73 | 1.86 | 0.13 | 0.00E+00 |
| VYZT01051392.1_272781 | gene body | 1414 | Cyp2j2 | 1.67 | -0.71 | 0.96 | 1.78E-07 |
| VYZT01055118.1_53001 | gene body | 52346 | Dab2ip | 1.90 | 0.20 | 2.10 | 4.34E-03 |
| VYZT01015243.1_60408 | gene body | 11890 | Dchs1 | -1.13 | 0.41 | -0.73 | 4.43E-34 |
| VYZT01004208.1_246257 | gene body | 22009 | Dennd1a | 1.95 | 0.75 | 2.69 | 6.98E-03 |
| VYZT01005454.1_87885 | gene body | 7280 | Dhx38 | 1.71 | 0.65 | 2.36 | 4.89E-03 |
| VYZT01000034.1_41577 | gene body | 24121 | Dlg3 | 4.99 | -0.15 | 4.84 | 6.51E-05 |
| VYZT01000034.1_47670 | gene body | 18028 | Dlg3 | 1.60 | 0.37 | 1.97 | 4.36E-03 |
| VYZT01000034.1_40444 | gene body | 25254 | Dlg3 | 2.22 | 0.52 | 2.74 | 2.23E-03 |
| VYZT01061371.1_33927 | gene body | 5025 | Dll1 | 2.24 | -0.02 | 2.22 | 5.17E-03 |
| VYZT01055535.1_254303 | gene body | -14985 | Dusp28 | 1.90 | 0.50 | 2.41 | 7.39E-03 |
| VYZT01033043.1_115201 | gene body | 4219 | E2f4 | 2.17 | 0.75 | 2.92 | 1.72E-03 |
| VYZT01055559.1_17563 | gene body | 508 | Ebi3 | 1.97 | 0.68 | 2.66 | 7.51E-03 |
| VYZT01020468.1_190027 | gene body | 13566 | Ehd3 | 6.52 | -1.29 | 5.23 | 6.02E-14 |
| VYZT01026691.1_19381 | gene body | 7221 | Eif2d | 2.53 | 0.93 | 3.46 | 1.31E-04 |
| VYZT01013754.1_504494 | gene body | 166953 | Elfn1 | -1.87 | 2.49 | 0.61 | 9.86E-03 |
| VYZT01062735.1_364239 | gene body | 5048 | Epha4 | 1.59 | 0.08 | 1.66 | 5.82E-03 |
| VYZT01062735.1_364227 | gene body | 5060 | Epha4 | 2.40 | 0.43 | 2.83 | 2.93E-04 |
| VYZT01060198.1_34153 | gene body | 8232 | Etf1 | 2.02 | 0.82 | 2.84 | 6.31E-03 |
| VYZT01063172.1_52782 | gene body | 2923 | Etv5 | 2.56 | -1.54 | 1.02 | 3.61E-09 |
| VYZT01008907.1_167551 | gene body | 2863 | Ext1 | 2.25 | 0.69 | 2.94 | 7.27E-03 |
| VYZT01016129.1_5644 | gene body | 3690 | Faim2 | 1.62 | -0.13 | 1.48 | 1.17E-117 |
| VYZT01062728.1_144845 | gene body | 27933 | Fam19a4 | 2.55 | 0.84 | 3.39 | 7.61E-04 |
| VYZT01039684.1_10016 | gene body | 9830 | Fam96a | 1.34 | -0.01 | 1.32 | 1.47E-06 |
| VYZT01043235.1_62253 | gene body | 9991 | Fanca | 2.21 | 0.85 | 3.07 | 3.01E-03 |
| VYZT01003555.1_4760 | gene body | 12745 | Fer1l4 | -2.13 | 2.68 | 0.55 | 9.42E-03 |
| VYZT01008402.1_138596 | gene body | 14204 | Fgfr2 | 1.73 | -0.36 | 1.37 | 1.99E-04 |
| VYZT01023774.1_23712 | gene body | -18614 | Gatad1 | 2.08 | 0.54 | 2.62 | 6.30E-03 |
| VYZT01033764.1_27384 | gene body | 5038 | Gbgt1 | 1.80 | 0.62 | 2.42 | 7.97E-03 |
| VYZT01032656.1_131926 | gene body | 3465 | Gcgr | 2.21 | 0.80 | 3.01 | 9.53E-04 |
| VYZT01000308.1_36281 | gene body | 3075 | Gfod2 | 2.02 | 0.64 | 2.66 | 1.31E-03 |
| VYZT01014681.1_135149 | gene body | 52339 | Ghsr | 2.31 | 0.76 | 3.08 | 6.01E-04 |
| VYZT01060714.1_67288 | gene body | 1109 | Gipc3 | 1.10 | -0.12 | 0.98 | 2.47E-13 |
| VYZT01024121.1_329433 | gene body | -22630 | Gphb5 | 1.77 | 0.38 | 2.15 | 8.16E-03 |
| VYZT01007502.1_61201 | gene body | 615 | Gpr19 | 1.96 | 0.26 | 2.22 | 8.95E-03 |
| VYZT01004208.1_83209 | gene body | 3898 | Gpr21 | 2.00 | 0.34 | 2.34 | 1.59E-04 |
| VYZT01056688.1_292651 | gene body | 3705 | Grik1 | 2.41 | -0.17 | 2.24 | 2.38E-04 |
| VYZT01054217.1_103809 | gene body | 4539 | Grin1 | 2.20 | 0.60 | 2.81 | 2.63E-03 |
| VYZT01022310.1_167381 | gene body | 28717 | Grm7 | 2.13 | -0.08 | 2.05 | 2.29E-03 |
| VYZT01022060.1_9468 | gene body | 817 | Grn | -2.39 | 0.96 | -1.43 | 1.38E-13 |
| VYZT01035962.1_345457 | gene body | 6655 | Gtf3c1 | 2.05 | 0.73 | 2.78 | 3.37E-03 |
| VYZT01009702.1_42362 | gene body | 1060 | Hapln3 | 2.11 | 0.40 | 2.52 | 3.80E-03 |
| VYZT01030577.1_29814 | gene body | 6326 | Has2 | 2.27 | 0.33 | 2.60 | 8.29E-03 |
| VYZT01018756.1_43769 | gene body | 19118 | Hdac4 | 1.41 | 0.23 | 1.64 | 1.81E-25 |
| VYZT01019215.1_264243 | gene body | 4019 | Higd2a | 1.83 | 0.74 | 2.57 | 9.23E-03 |
| VYZT01019215.1_357972 | gene body | 2501 | Hk2 | 1.86 | 0.73 | 2.58 | 5.59E-03 |
| VYZT01065321.1_641091 | gene body | 25376 | Hlf | 1.32 | -0.37 | 0.95 | 1.02E-20 |
| VYZT01058839.1_30855 | gene body | 2002 | Hmgcl | 2.53 | 0.38 | 2.90 | 2.13E-03 |
| VYZT01063215.1_2893 | gene body | 2875 | Hnf4b | -6.38 | 5.29 | -1.09 | 0.00E+00 |
| VYZT01034596.1_2791 | gene body | 1106 | Hspbap1 | 2.09 | 0.65 | 2.74 | 2.75E-03 |
| VYZT01028664.1_15739 | gene body | 8798 | Ift81 | 1.67 | -0.07 | 1.61 | 1.44E-22 |
| VYZT01012492.1_128644 | gene body | 848 | Il10ra | -1.91 | 2.61 | 0.70 | 7.51E-03 |
| VYZT01028644.1_191201 | gene body | 16326 | Impg2 | 1.97 | 0.68 | 2.65 | 9.98E-03 |
| VYZT01058133.1_395739 | gene body | 7858 | Iqgap3 | 1.96 | 0.78 | 2.74 | 6.20E-03 |
| VYZT01007622.1_4451 | gene body | 17952 | Iqsec1 | -2.22 | 2.98 | 0.77 | 6.94E-04 |
| VYZT01040865.1_74735 | gene body | 45763 | Itga9 | 2.35 | 0.37 | 2.72 | 2.48E-03 |
| VYZT01033104.1_40131 | gene body | 27527 | Kank4 | 1.82 | 0.63 | 2.45 | 7.18E-03 |
| VYZT01047449.1_90792 | gene body | 33223 | Kcnk10 | 2.00 | 0.59 | 2.59 | 2.47E-03 |
| VYZT01025028.1_73954 | gene body | 53596 | Kif26a | 3.03 | -0.39 | 2.65 | 6.68E-43 |
| VYZT01015840.1_200621 | gene body | 2944 | Kpna1 | 1.95 | 0.73 | 2.68 | 4.04E-03 |
| VYZT01047499.1_11136 | gene body | 207 | Krsc | 1.30 | -0.06 | 1.24 | 2.48E-03 |
| VYZT01018718.1_294256 | gene body | 4164 | Larp1 | 1.81 | 0.29 | 2.09 | 7.65E-03 |
| VYZT01034503.1_323790 | gene body | 13061 | Lars | 1.09 | -0.35 | 0.74 | 1.14E-03 |
| VYZT01025783.1_64722 | gene body | 2570 | Lipm | -1.29 | 1.48 | 0.19 | 0.00E+00 |
| VYZT01037706.1_74764 | gene body | 18111 | Llgl2 | 2.24 | -0.58 | 1.66 | 2.06E-04 |
| VYZT01047064.1_101156 | gene body | 10701 | Lonp2 | 2.58 | 0.88 | 3.46 | 1.32E-04 |
| VYZT01054913.1_179595 | gene body | 172824 | Lpp | -2.09 | 2.83 | 0.74 | 8.81E-04 |
| VYZT01002682.1_228603 | gene body | 94012 | Lrp6 | 2.32 | 0.88 | 3.20 | 9.83E-04 |
| VYZT01005471.1_10588 | gene body | 5596 | Lrrc10b | 2.05 | 0.77 | 2.82 | 8.71E-03 |
| VYZT01061633.1_44501 | gene body | 755 | Lrrc55 | 2.07 | 0.76 | 2.82 | 4.97E-03 |
| VYZT01005938.1_55318 | gene body | -12157 | Lztr1 | 2.16 | 0.70 | 2.86 | 7.97E-03 |
| VYZT01033714.1_103174 | gene body | 38498 | Map2k4 | 2.09 | 0.74 | 2.83 | 3.30E-03 |
| VYZT01035980.1_101825 | gene body | 6890 | Mast3 | -2.14 | 2.96 | 0.82 | 8.14E-03 |
| VYZT01028128.1_32586 | gene body | 5854 | Med19 | -2.22 | 2.76 | 0.55 | 3.81E-03 |
| VYZT01060106.1_78040 | gene body | 30763 | Meis1 | 1.98 | 0.09 | 2.08 | 3.60E-111 |
| VYZT01000771.1_104629 | gene body | 35921 | Met | 2.34 | 0.88 | 3.22 | 2.33E-03 |
| VYZT01029205.1_154413 | gene body | -3443 | Mfn2 | 2.08 | 0.56 | 2.64 | 9.01E-03 |
| VYZT01040755.1_25288 | gene body | -5059 | Mfrp | -2.24 | 3.07 | 0.83 | 6.64E-03 |
| VYZT01005496.1_27126 | gene body | 13471 | Mfsd10 | 1.79 | -0.03 | 1.76 | 1.28E-08 |
| VYZT01005975.1_88995 | gene body | 2245 | Mgat4b | 1.95 | 0.57 | 2.52 | 1.82E-03 |
| VYZT01025839.1_38021 | gene body | 21617 | Mme | 2.16 | 0.02 | 2.18 | 8.03E-03 |
| VYZT01020442.1_28638 | gene body | 24950 | Mmp24 | 2.64 | 0.68 | 3.31 | 9.70E-05 |
| VYZT01011173.1_30817 | gene body | 1980 | Mospd1 | 2.48 | 0.82 | 3.31 | 9.92E-04 |
| VYZT01028657.1_107224 | gene body | 22187 | Mtmr4 | 2.08 | 0.64 | 2.72 | 1.15E-03 |
| VYZT01040776.1_44052 | gene body | -11280 | Mtr | -2.16 | 2.89 | 0.74 | 3.32E-03 |
| VYZT01014608.1_132301 | gene body | 8139 | Mxd4 | 1.61 | 0.39 | 2.01 | 7.06E-03 |
| VYZT01045530.1_30028 | gene body | 1138 | Myh1b | 2.64 | 0.77 | 3.41 | 1.56E-04 |
| VYZT01025807.1_52946 | gene body | 25078 | Myo10 | 1.90 | 0.13 | 2.04 | 3.78E-07 |
| VYZT01009684.1_141285 | gene body | 11274 | Myog | -1.91 | 2.56 | 0.65 | 8.76E-04 |
| VYZT01014178.1_24478 | gene body | 22642 | Nat8l | 1.55 | 0.51 | 2.06 | 8.95E-03 |
| VYZT01048850.1_79255 | gene body | 8797 | Nckap5 | -1.91 | 2.24 | 0.33 | 0.00E+00 |
| VYZT01022834.1_60131 | gene body | 2346 | Ndnf | 2.18 | 0.48 | 2.66 | 1.22E-03 |
| VYZT01052123.1_18998 | gene body | 17374 | Nf2 | 2.28 | 0.25 | 2.54 | 1.63E-03 |
| VYZT01001097.1_40579 | gene body | 3108 | Nfkb2 | 1.84 | 0.42 | 2.25 | 7.33E-03 |
| VYZT01058553.1_75762 | gene body | 1209 | Ngef | -1.34 | 1.31 | -0.03 | 6.92E-55 |
| VYZT01043808.1_175730 | gene body | 489 | Nhsl2 | 1.97 | 0.77 | 2.74 | 3.17E-03 |
| VYZT01043808.1_168318 | gene body | -6923 | Nhsl2 | 1.30 | 0.14 | 1.44 | 5.52E-14 |
| VYZT01021899.1_9066 | gene body | 2956 | Nmrk2 | 2.59 | 0.54 | 3.13 | 6.45E-06 |
| VYZT01005923.1_124146 | gene body | 37714 | Noc2l | 1.93 | 0.71 | 2.64 | 7.50E-03 |
| VYZT01043122.1_17706 | gene body | 1403 | Nop56 | 1.70 | 0.31 | 2.00 | 8.49E-03 |
| VYZT01005383.1_6896 | gene body | 1059 | Npepps | 2.31 | 0.67 | 2.98 | 2.22E-03 |
| VYZT01036557.1_144012 | gene body | 3508 | Nr1d1 | 1.59 | 0.28 | 1.88 | 7.11E-15 |
| VYZT01022842.1_94693 | gene body | 92428 | Nrg3 | 2.23 | 0.81 | 3.04 | 6.17E-03 |
| VYZT01007607.1_294765 | gene body | 4760 | Nsun4 | -1.98 | 2.74 | 0.76 | 4.66E-03 |
| VYZT01009702.1_444663 | gene body | 180800 | Ntrk3 | -1.14 | 0.96 | -0.18 | 0.00E+00 |
| VYZT01009702.1_464670 | gene body | 200807 | Ntrk3 | -3.02 | 3.99 | 0.98 | 1.04E-06 |
| VYZT01015779.1_6953 | gene body | 8003 | Oat | -2.12 | 2.87 | 0.75 | 8.71E-03 |
| VYZT01060709.1_63636 | gene body | 7776 | Opn5 | 1.99 | 0.50 | 2.50 | 1.99E-03 |
| VYZT01057616.1_206867 | gene body | -18125 | Opsp | -2.24 | 2.98 | 0.74 | 1.80E-03 |
| VYZT01028664.1_215403 | gene body | 504 | Orai1 | 1.52 | 0.08 | 1.60 | 9.41E-03 |
| VYZT01062722.1_76166 | gene body | -4916 | Osbp | 1.90 | 0.68 | 2.58 | 6.00E-03 |
| VYZT01062722.1_116694 | gene body | 21366 | Osbp2 | 2.26 | 0.46 | 2.72 | 7.38E-03 |
| VYZT01018090.1_149256 | gene body | 29580 | Pacs2 | -2.07 | 2.79 | 0.72 | 4.85E-04 |
| VYZT01042208.1_120356 | gene body | 33199 | Pappa | 2.00 | 0.30 | 2.30 | 1.57E-03 |
| VYZT01042208.1_40807 | gene body | 112748 | Pappa | 1.89 | -0.31 | 1.58 | 5.12E-04 |
| VYZT01065376.1_42777 | gene body | 298 | Pars2 | 2.31 | 0.90 | 3.21 | 2.41E-04 |
| VYZT01013442.1_197297 | gene body | 18461 | Pbx1 | 1.90 | 0.16 | 2.07 | 1.28E-03 |
| VYZT01046940.1_42158 | gene body | 5077 | Phlda2 | 2.12 | -0.21 | 1.91 | 2.47E-06 |
| VYZT01038371.1_4297 | gene body | 2824 | Pik3c2b | 2.07 | 0.79 | 2.86 | 2.85E-03 |
| VYZT01014728.1_16192 | gene body | 2472 | Pla2g4e | 2.28 | 0.77 | 3.05 | 9.17E-04 |
| VYZT01027224.1_48470 | gene body | 21135 | Plcg1 | 1.98 | 0.80 | 2.78 | 4.41E-03 |
| VYZT01001285.1_5423 | gene body | 1483 | Plekhb1 | 2.16 | 0.83 | 2.99 | 4.38E-03 |
| VYZT01039015.1_32210 | gene body | 26066 | Plxna2 | 2.01 | 0.71 | 2.72 | 6.30E-03 |
| VYZT01002930.1_8260 | gene body | 6172 | Pole2 | 1.93 | 0.73 | 2.66 | 7.28E-03 |
| VYZT01048762.1_36134 | gene body | 17879 | Ppfibp2 | -2.29 | 3.15 | 0.86 | 2.13E-03 |
| VYZT01014611.1_45036 | gene body | 8384 | Ppm1g | 2.38 | -0.34 | 2.04 | 7.82E-03 |
| VYZT01018194.1_167220 | gene body | 4771 | Ppme1 | 4.25 | 0.59 | 4.84 | 4.62E-03 |
| VYZT01036184.1_110298 | gene body | 14705 | Pqlc1 | 1.40 | 0.17 | 1.57 | 0.00E+00 |
| VYZT01008882.1_249697 | gene body | 19059 | Prickle2 | -2.77 | 3.67 | 0.90 | 6.79E-04 |
| VYZT01008882.1_200840 | gene body | -29798 | Prickle2 | 1.24 | 0.24 | 1.48 | 2.04E-03 |
| VYZT01014659.1_18301 | gene body | -10215 | Prkag3 | 2.60 | 0.21 | 2.81 | 8.10E-08 |
| VYZT01008882.1_315273 | gene body | -49007 | Psmd6 | 1.97 | 0.79 | 2.76 | 8.60E-04 |
| VYZT01028168.1_25643 | gene body | 17417 | Psmg4 | 1.11 | -0.26 | 0.85 | 6.32E-67 |
| VYZT01060310.1_100018 | gene body | 23745 | Pstpip1 | 1.36 | 0.00 | 1.35 | 1.47E-16 |
| VYZT01001928.1_24724 | gene body | 8741 | Ptpa | 2.05 | -0.12 | 1.93 | 7.46E-03 |
| VYZT01026106.1_107826 | gene body | 80584 | Ptprf | 2.02 | 0.73 | 2.75 | 3.31E-03 |
| VYZT01035272.1_431039 | gene body | 39336 | Ptprs | 2.16 | 0.27 | 2.42 | 9.36E-03 |
| VYZT01044761.1_40730 | gene body | 8989 | Ptpru | 1.85 | 0.63 | 2.48 | 9.09E-03 |
| VYZT01037385.1_50260 | gene body | 13095 | Ptprv | 2.37 | 0.42 | 2.79 | 2.24E-03 |
| VYZT01035327.1_168721 | gene body | -12665 | Pus1 | 1.86 | 0.52 | 2.38 | 6.86E-03 |
| VYZT01032994.1_9099 | gene body | 9095 | Pygb | 2.69 | 0.58 | 3.27 | 2.51E-94 |
| VYZT01025776.1_6879 | gene body | 5848 | Pygl | 2.04 | 0.56 | 2.60 | 8.48E-03 |
| VYZT01063199.1_106286 | gene body | -45665 | Rassf10 | 1.81 | 0.63 | 2.44 | 9.65E-03 |
| VYZT01011226.1_17393 | gene body | 5655 | Rbpjl | 2.42 | 0.75 | 3.16 | 8.98E-04 |
| VYZT01036557.1_23930 | gene body | 7163 | Rdh8 | 2.27 | 0.84 | 3.11 | 3.39E-03 |
| VYZT01014596.1_141143 | gene body | 53975 | Rflna | 8.26 | -5.58 | 2.68 | 1.91E-144 |
| VYZT01067014.1_7431 | gene body | 8844 | Rgmb | -1.95 | 2.73 | 0.78 | 5.07E-03 |
| VYZT01016085.1_121331 | gene body | 43780 | Rgs3 | 1.51 | -0.53 | 0.99 | 2.56E-03 |
| VYZT01016085.1_102420 | gene body | 24869 | Rgs3 | 1.64 | -1.29 | 0.35 | 7.90E-38 |
| VYZT01018639.1_19130 | gene body | 17356 | Rhbdl3 | 1.59 | 0.48 | 2.08 | 2.22E-03 |
| VYZT01000294.1_11525 | gene body | 6455 | Rhcg | 2.31 | 0.56 | 2.87 | 5.49E-04 |
| VYZT01028664.1_106055 | gene body | 19877 | Rnf34 | 1.78 | -0.37 | 1.41 | 3.87E-03 |
| VYZT01011847.1_54816 | gene body | 12784 | Rnl2 | 2.44 | -0.80 | 1.64 | 1.29E-23 |
| VYZT01001425.1_23107 | gene body | -16950 | Rps17 | 1.73 | -0.43 | 1.31 | 9.61E-03 |
| VYZT01003329.1_166872 | gene body | 2008 | Rspo1 | 1.81 | 0.64 | 2.45 | 4.87E-03 |
| VYZT01057662.1_23022 | gene body | 26051 | Rspo3 | -2.76 | 3.56 | 0.80 | 3.04E-05 |
| VYZT01021101.1_64459 | gene body | 7058 | Sap30bp | 1.60 | 0.16 | 1.77 | 2.47E-03 |
| VYZT01009690.1_63138 | gene body | 9259 | Sardh | 2.27 | -0.10 | 2.18 | 1.78E-03 |
| VYZT01054862.1_49000 | gene body | 7044 | Sash3 | 2.34 | 0.89 | 3.22 | 2.26E-04 |
| VYZT01063558.1_40945 | gene body | 6275 | Scaf11 | 1.49 | 0.06 | 1.56 | 9.69E-03 |
| VYZT01032166.1_7102 | gene body | 17557 | Scube3 | 2.09 | 0.34 | 2.43 | 1.30E-03 |
| VYZT01037108.1_53512 | gene body | -26488 | Sec16a | 3.23 | -1.65 | 1.57 | 7.84E-12 |
| VYZT01052191.1_282570 | gene body | 4095 | Sec24d | 1.77 | 0.37 | 2.14 | 0.00E+00 |
| VYZT01023625.1_239499 | gene body | 570 | Selp | 1.94 | 0.71 | 2.66 | 8.65E-03 |
| VYZT01039009.1_190627 | gene body | 36575 | Sept9 | 1.25 | -0.01 | 1.24 | 3.11E-03 |
| VYZT01063220.1_954538 | gene body | 987 | Sh2d3c | 1.58 | 0.07 | 1.66 | 5.67E-68 |
| VYZT01022851.1_335469 | gene body | 3289 | Sik3 | 1.71 | 0.44 | 2.15 | 0.00E+00 |
| VYZT01022851.1_361585 | gene body | 4084 | Sik3 | 2.19 | -0.24 | 1.95 | 1.98E-06 |
| VYZT01013295.1_31625 | gene body | 6556 | Slail | 2.09 | 0.83 | 2.92 | 9.44E-03 |
| VYZT01017233.1_46574 | gene body | 2678 | Slc2a6 | 2.05 | 0.54 | 2.60 | 2.70E-03 |
| VYZT01024965.1_523991 | gene body | 21457 | Slc36a1 | 2.14 | 0.69 | 2.83 | 2.82E-03 |
| VYZT01041525.1_203695 | gene body | 5351 | Slc38a3 | -2.03 | 2.27 | 0.25 | 0.00E+00 |
| VYZT01034657.1_67264 | gene body | 5800 | Slc4a3 | -1.98 | 2.75 | 0.76 | 5.40E-03 |
| VYZT01059599.1_10783 | gene body | 1786 | Slc6a4 | 1.93 | 0.76 | 2.68 | 3.72E-03 |
| VYZT01003403.1_87648 | gene body | 31790 | Slc8a3 | 2.14 | 0.26 | 2.40 | 1.71E-03 |
| VYZT01009668.1_146401 | gene body | 5420 | Slc9a1 | -2.02 | 2.74 | 0.72 | 6.69E-03 |
| VYZT01056688.1_333049 | gene body | -29649 | Slco2b1 | 2.04 | 0.56 | 2.61 | 5.16E-03 |
| VYZT01002017.1_35144 | gene body | 1467 | Slitrk1 | 1.97 | 0.60 | 2.56 | 6.97E-03 |
| VYZT01023570.1_112205 | gene body | 921 | Slitrk2 | 1.86 | 0.14 | 2.00 | 4.30E-03 |
| VYZT01059371.1_67541 | gene body | 4912 | Smad3 | -1.40 | 1.68 | 0.29 | 0.00E+00 |
| VYZT01019165.1_269017 | gene body | 11970 | Smyd1 | 1.53 | 0.32 | 1.85 | 5.02E-232 |
| VYZT01004753.1_14068 | gene body | 5806 | Snta1 | 2.62 | -1.12 | 1.51 | 2.40E-03 |
| VYZT01052886.1_1272 | gene body | 507 | Socs3 | 2.19 | 0.53 | 2.72 | 1.32E-04 |
| VYZT01060110.1_106917 | gene body | 71387 | Spata5 | 2.18 | 0.79 | 2.96 | 3.50E-03 |
| VYZT01027281.1_41618 | gene body | 6331 | Spen | 1.85 | 0.66 | 2.51 | 4.21E-03 |
| VYZT01027330.1_59601 | gene body | 20845 | Spg7 | 6.26 | -0.68 | 5.58 | 1.84E-48 |
| VYZT01027330.1_55637 | gene body | 16881 | Spg7 | 1.80 | 0.51 | 2.31 | 4.05E-03 |
| VYZT01048233.1_516145 | gene body | 3092 | Spopl | 3.28 | 0.61 | 3.88 | 1.53E-06 |
| VYZT01012434.1_194509 | gene body | 5270 | Srebf1 | 1.88 | 0.59 | 2.47 | 5.87E-03 |
| VYZT01013214.1_51126 | gene body | 3410 | Stim1 | 2.78 | 0.26 | 3.04 | 1.38E-03 |
| VYZT01026558.1_74423 | gene body | 5099 | Stom | 2.07 | 0.28 | 2.35 | 6.99E-54 |
| VYZT01063294.1_2889 | gene body | 2580 | Svop | 2.00 | 0.45 | 2.45 | 8.92E-03 |
| VYZT01063294.1_2878 | gene body | 2569 | Svop | 2.73 | -0.52 | 2.22 | 6.82E-04 |
| VYZT01050763.1_128455 | gene body | 12718 | Syt12 | 1.77 | -0.37 | 1.40 | 7.08E-03 |
| VYZT01010488.1_1079 | gene body | 2423 | Tars | 2.00 | 0.19 | 2.20 | 8.86E-03 |
| VYZT01032987.1_20223 | gene body | 20196 | Tbx5 | 1.67 | 0.50 | 2.17 | 4.73E-03 |
| VYZT01023625.1_576620 | gene body | 56247 | Tedc1 | 2.15 | 0.65 | 2.81 | 4.44E-03 |
| VYZT01063892.1_341096 | gene body | 3212 | Tesk2 | 2.11 | 0.85 | 2.96 | 3.16E-03 |
| VYZT01031188.1_317630 | gene body | 35179 | Tet1 | 2.36 | 0.39 | 2.75 | 4.42E-03 |
| VYZT01046247.1_58087 | gene body | 3665 | Tjp3 | 1.81 | 0.72 | 2.53 | 5.17E-03 |
| VYZT01062002.1_86688 | gene body | -3966 | Tmem41a | 2.98 | 0.92 | 3.90 | 3.66E-08 |
| VYZT01034751.1_9893 | gene body | -3298 | Tnfrsf25 | 2.31 | 0.39 | 2.69 | 2.80E-03 |
| VYZT01014634.1_169854 | gene body | 260 | Tnr | 1.95 | 0.36 | 2.30 | 4.28E-03 |
| VYZT01012434.1_156969 | gene body | 13107 | Tom1l2 | 2.25 | 0.10 | 2.35 | 1.86E-03 |
| VYZT01012434.1_166257 | gene body | 22395 | Tom1l2 | 2.22 | 0.87 | 3.09 | 2.11E-03 |
| VYZT01044766.1_108417 | gene body | 232 | Tprn | 1.99 | 0.62 | 2.61 | 8.16E-03 |
| VYZT01021147.1_6277 | gene body | 5181 | Trmt6 | 2.05 | 0.39 | 2.44 | 4.39E-03 |
| VYZT01060151.1_11272 | gene body | 1532 | Trpm2 | 1.55 | 0.04 | 1.60 | 8.96E-03 |
| VYZT01029293.1_221787 | gene body | -6300 | Ttc36 | 2.80 | 0.70 | 3.50 | 5.81E-06 |
| VYZT01013225.1_49160 | gene body | 2022 | Ttyh2 | 1.99 | 0.34 | 2.33 | 3.80E-03 |
| VYZT01028644.1_132099 | gene body | 6204 | Ubfd1 | 2.20 | 0.77 | 2.97 | 9.98E-03 |
| VYZT01009747.1_104806 | gene body | -59303 | Vamp3 | 1.75 | -0.65 | 1.10 | 0.00E+00 |
| VYZT01034508.1_57686 | gene body | 38503 | Wac | 1.44 | 0.29 | 1.73 | 2.41E-03 |
| VYZT01045448.1_16580 | gene body | 937 | Zbtb14 | 1.67 | 0.03 | 1.70 | 3.16E-03 |
| VYZT01029824.1_113516 | gene body | 75431 | Zfhx3 | 2.33 | 0.85 | 3.18 | 1.77E-03 |
| VYZT01019215.1_405020 | gene body | 2763 | Znf346 | 1.62 | 0.35 | 1.98 | 6.76E-80 |
| VYZT01002030.1_82347 | gene body | 12117 | Znf385c | 1.33 | -0.23 | 1.10 | 6.34E-36 |
| VYZT01041703.1_383162 | gene body | 97722 | Znf423 | 1.52 | 0.10 | 1.62 | 0.00E+00 |
| VYZT01015144.1_36823 | upstream10k | -4533 | Acvr1b | 1.77 | 0.63 | 2.40 | 8.32E-03 |
| VYZT01025100.1_80265 | upstream10k | -5852 | Agap3 | 1.42 | 0.17 | 1.59 | 9.89E-06 |
| VYZT01048789.1_25187 | upstream10k | -6189 | Atp6v1g1 | 3.24 | 0.33 | 3.57 | 5.98E-55 |
| VYZT01015151.1_133431 | upstream10k | -3381 | Atrx | 1.80 | -0.19 | 1.61 | 2.47E-03 |
| VYZT01014099.1_49962 | upstream10k | -4805 | Avpr1b | 2.60 | 0.85 | 3.44 | 4.74E-04 |
| VYZT01009750.1_200233 | upstream10k | -6020 | Bank1 | 1.58 | 0.09 | 1.67 | 8.18E-03 |
| VYZT01047650.1_30202 | upstream10k | -4795 | Bmp7 | 2.10 | 0.62 | 2.73 | 6.60E-03 |
| VYZT01026373.1_41208 | upstream10k | -8205 | Boc | 2.18 | 0.62 | 2.80 | 4.76E-03 |
| VYZT01051102.1_1813 | upstream10k | -8504 | Bod1 | -2.34 | 3.13 | 0.79 | 1.39E-03 |
| VYZT01000689.1_44958 | upstream10k | -2282 | Bricd5 | 3.45 | -1.16 | 2.29 | 1.02E-07 |
| VYZT01055665.1_199541 | upstream10k | -9299 | Cadps | 2.02 | 0.61 | 2.63 | 9.27E-03 |
| VYZT01034534.1_17662 | upstream10k | -8424 | Cdc16 | 2.26 | -1.02 | 1.23 | 2.31E-34 |
| VYZT01063217.1_38924 | upstream10k | -9636 | Cdc42ep4 | 2.46 | 0.75 | 3.21 | 9.34E-76 |
| VYZT01022851.1_453012 | upstream10k | 1071 | Cep164 | 1.94 | 0.37 | 2.31 | 3.58E-03 |
| VYZT01022845.1_153311 | upstream10k | -2608 | Chd4 | 1.39 | -0.65 | 0.74 | 4.90E-03 |
| VYZT01011795.1_204573 | upstream10k | -2411 | Chrd | 1.66 | 0.39 | 2.05 | 7.77E-14 |
| VYZT01022356.1_257464 | upstream10k | -8047 | Chst14 | 1.88 | 0.56 | 2.44 | 7.38E-03 |
| VYZT01057572.1_63159 | upstream10k | 3277 | Cib1 | 1.82 | 0.29 | 2.11 | 6.01E-03 |
| VYZT01047541.1_57830 | upstream10k | -2389 | Cmtm4 | 1.83 | 0.64 | 2.47 | 9.11E-03 |
| VYZT01032561.1_68046 | upstream10k | -8073 | Cnksr2 | 1.58 | 0.06 | 1.65 | 5.42E-03 |
| VYZT01010505.1_26949 | upstream10k | 4007 | Cyp1a5 | 2.01 | 0.36 | 2.37 | 2.94E-03 |
| VYZT01043817.1_59642 | upstream10k | -4689 | Cyth1 | 1.84 | 0.59 | 2.43 | 3.89E-03 |
| VYZT01000602.1_182364 | upstream10k | -7455 | Drp2 | 1.46 | -0.16 | 1.30 | 5.15E-03 |
| VYZT01017207.1_98763 | upstream10k | -3472 | Erich1 | 1.92 | 0.43 | 2.35 | 6.65E-03 |
| VYZT01036688.1_135187 | upstream10k | -9000 | Etfa | -2.16 | 2.92 | 0.76 | 3.50E-03 |
| VYZT01054145.1_11752 | upstream10k | -2416 | Etfrf1 | 1.70 | 0.59 | 2.29 | 8.24E-03 |
| VYZT01011868.1_13757 | upstream10k | -8768 | Faah2 | 2.29 | 0.12 | 2.41 | 2.23E-03 |
| VYZT01010580.1_19263 | upstream10k | -6099 | Fam187a | 1.17 | -0.55 | 0.62 | 0.00E+00 |
| VYZT01052924.1_79747 | upstream10k | 4592 | Fam78b | -1.96 | 2.64 | 0.68 | 8.68E-03 |
| VYZT01024198.1_57744 | upstream10k | -6376 | Frmd6 | 2.43 | 0.28 | 2.71 | 2.65E-102 |
| VYZT01024984.1_126426 | upstream10k | -9349 | Fyco1 | 2.28 | 0.77 | 3.05 | 6.91E-03 |
| VYZT01062670.1_94881 | upstream10k | -4628 | Galm | -2.98 | 3.77 | 0.79 | 9.07E-07 |
| VYZT01043130.1_148383 | upstream10k | -7237 | Gfra4 | 1.42 | -0.28 | 1.14 | 8.41E-03 |
| VYZT01063190.1_27704 | upstream10k | -8993 | Ggt7 | 1.45 | -0.51 | 0.94 | 1.97E-04 |
| VYZT01015256.1_44448 | upstream10k | -6280 | Gpr12 | 1.86 | 0.61 | 2.47 | 3.75E-16 |
| VYZT01063892.1_173021 | upstream10k | -2796 | Hectd3 | 1.91 | 0.56 | 2.48 | 2.27E-03 |
| VYZT01027234.1_10531 | upstream10k | -5367 | Hectd4 | 2.17 | 0.81 | 2.98 | 8.42E-03 |
| VYZT01041846.1_58338 | upstream10k | -8154 | Kazald1 | 1.91 | 0.56 | 2.47 | 6.87E-03 |
| VYZT01054245.1_64910 | upstream10k | -6744 | Kbtbd11 | 2.03 | 0.50 | 2.53 | 3.46E-04 |
| VYZT01030591.1_217469 | upstream10k | -8893 | Kcnh6 | 2.07 | 0.82 | 2.90 | 4.65E-03 |
| VYZT01040153.1_22512 | upstream10k | -7366 | Krt80 | 2.49 | 0.52 | 3.01 | 2.53E-03 |
| VYZT01040860.1_18828 | upstream10k | -5935 | Lrp1 | 2.53 | 0.81 | 3.35 | 9.19E-06 |
| VYZT01013754.1_842464 | upstream10k | -4448 | Mafk | 2.02 | 0.66 | 2.68 | 1.57E-03 |
| VYZT01047523.1_4828 | upstream10k | -2385 | Mbd3 | 8.63 | -3.78 | 4.85 | 2.56E-51 |
| VYZT01045109.1_82796 | upstream10k | -4984 | Mettl7a | 1.97 | 0.80 | 2.77 | 5.37E-03 |
| VYZT01006611.1_45885 | upstream10k | -2996 | Mga | 2.08 | 0.35 | 2.43 | 8.83E-03 |
| VYZT01020523.1_153418 | upstream10k | -7647 | Micalcl | 2.25 | 0.78 | 3.03 | 6.10E-04 |
| VYZT01062231.1_18333 | upstream10k | 5884 | Mocs3 | 2.01 | -0.14 | 1.86 | 2.90E-03 |
| VYZT01009684.1_155522 | upstream10k | -2963 | Myog | 1.84 | 0.45 | 2.29 | 6.47E-03 |
| VYZT01028640.1_26390 | upstream10k | -8122 | Myrf | 2.23 | 0.86 | 3.09 | 9.29E-05 |
| VYZT01005983.1_572163 | upstream10k | -4892 | Nipal4 | 1.66 | 0.37 | 2.03 | 5.03E-03 |
| VYZT01034771.1_31232 | upstream10k | -4963 | Nob1 | 1.74 | 0.41 | 2.14 | 4.33E-03 |
| VYZT01015816.1_285232 | upstream10k | 5704 | Nol12 | -2.63 | 3.41 | 0.78 | 1.42E-05 |
| VYZT01043122.1_24607 | upstream10k | -5498 | Nop56 | 2.00 | 0.77 | 2.77 | 4.95E-03 |
| VYZT01037207.1_195001 | upstream10k | -2873 | Ntn3 | 2.18 | 0.65 | 2.83 | 1.78E-03 |
| VYZT01064681.1_6338 | upstream10k | -3807 | Pcdhgc5 | 2.28 | 0.48 | 2.76 | 1.93E-03 |
| VYZT01047534.1_59511 | upstream10k | -6715 | Pdlim4 | 2.02 | 0.14 | 2.16 | 3.72E-22 |
| VYZT01054274.1_179053 | upstream10k | 2517 | Pex12 | 1.30 | -0.05 | 1.25 | 2.09E-28 |
| VYZT01064709.1_20851 | upstream10k | -7453 | Phactr4 | 1.97 | 0.65 | 2.62 | 3.74E-03 |
| VYZT01040995.1_48317 | upstream10k | -2655 | Phlda3 | 1.76 | 0.50 | 2.26 | 5.05E-03 |
| VYZT01000072.1_20288 | upstream10k | -3272 | Pias1 | 1.95 | 0.65 | 2.60 | 5.82E-03 |
| VYZT01053563.1_42890 | upstream10k | 2736 | Piezo1 | 6.81 | 0.31 | 7.12 | 1.73E-42 |
| VYZT01053563.1_42854 | upstream10k | 2700 | Piezo1 | 2.11 | 0.67 | 2.77 | 5.96E-03 |
| VYZT01050467.1_33865 | upstream10k | -3671 | Pigl | -2.43 | 2.84 | 0.42 | 2.61E-03 |
| VYZT01031185.1_29453 | upstream10k | -4706 | Plxna4 | 2.34 | 0.58 | 2.92 | 8.15E-03 |
| VYZT01001988.1_555023 | upstream10k | 1161 | Ppp1r3cb | -1.97 | 2.68 | 0.71 | 1.77E-03 |
| VYZT01064671.1_15458 | upstream10k | -4179 | Prelp | 2.61 | 0.43 | 3.04 | 8.46E-03 |
| VYZT01014659.1_26234 | upstream10k | -2282 | Prkag3 | 1.77 | 0.69 | 2.45 | 7.03E-03 |
| VYZT01018624.1_53662 | upstream10k | -4885 | Pten | -2.67 | 3.37 | 0.70 | 4.45E-05 |
| VYZT01022872.1_13950 | upstream10k | -2310 | Pwwp2b | 1.65 | 0.28 | 1.94 | 3.63E-03 |
| VYZT01022221.1_34499 | upstream10k | -5424 | Rab42 | 2.02 | 0.26 | 2.28 | 4.27E-03 |
| VYZT01001921.1_21158 | upstream10k | -2248 | Rabggtb | 2.14 | 0.50 | 2.64 | 4.66E-03 |
| VYZT01040776.1_12739 | upstream10k | -6656 | Rad18 | 1.98 | 0.46 | 2.44 | 2.85E-03 |
| VYZT01026477.1_52637 | upstream10k | -6254 | Rars | 1.89 | 0.48 | 2.37 | 1.99E-03 |
| VYZT01032598.1_12506 | upstream10k | -6724 | Rgma | -5.43 | 5.49 | 0.05 | 2.96E-06 |
| VYZT01017291.1_348603 | upstream10k | -4652 | Rhbdl1 | 1.99 | 0.56 | 2.55 | 7.74E-03 |
| VYZT01054217.1_45667 | upstream10k | -3471 | Rnf208 | 1.23 | 0.26 | 1.49 | 1.13E-11 |
| VYZT01062125.1_57031 | upstream10k | -7703 | Rnf213 | -2.63 | 3.55 | 0.92 | 1.54E-04 |
| VYZT01013225.1_35632 | upstream10k | -9210 | Rpl38 | 2.20 | 0.42 | 2.63 | 9.14E-03 |
| VYZT01043264.1_27854 | upstream10k | -6230 | Sall4 | 1.34 | 0.17 | 1.50 | 3.50E-06 |
| VYZT01005923.1_190615 | upstream10k | -7552 | Samd11 | 1.78 | 0.07 | 1.86 | 7.04E-04 |
| VYZT01063217.1_8567 | upstream10k | -3505 | Sdk2 | 1.66 | 0.53 | 2.20 | 5.55E-03 |
| VYZT01037108.1_29282 | upstream10k | -2258 | Sec16a | 1.85 | 0.68 | 2.52 | 3.37E-03 |
| VYZT01015910.1_120477 | upstream10k | -7865 | Sept4 | 1.88 | -0.40 | 1.47 | 7.37E-03 |
| VYZT01044428.1_22970 | upstream10k | -6949 | Sgpp2 | 3.25 | -1.15 | 2.10 | 0.00E+00 |
| VYZT01013284.1_210891 | upstream10k | -2041 | Sh2b2 | 2.16 | 0.85 | 3.01 | 4.02E-03 |
| VYZT01031181.1_92623 | upstream10k | -3166 | Sirt4 | 2.10 | 0.74 | 2.84 | 4.23E-03 |
| VYZT01040055.1_24496 | upstream10k | -2517 | Slc37a1 | 1.86 | -0.52 | 1.34 | 6.54E-03 |
| VYZT01046170.1_99183 | upstream10k | -2035 | Snrk | -1.88 | 2.13 | 0.25 | 1.87E-04 |
| VYZT01002768.1_8468 | upstream10k | -3147 | Sox9 | 1.45 | 0.18 | 1.63 | 0.00E+00 |
| VYZT01025818.1_20989 | upstream10k | -3716 | Spryd7 | 1.94 | -0.63 | 1.32 | 5.69E-03 |
| VYZT01013284.1_421329 | upstream10k | -9465 | Srrm3 | 2.12 | 0.84 | 2.96 | 4.46E-03 |
| VYZT01055576.1_123876 | upstream10k | -2441 | Srsf6 | 1.65 | -0.05 | 1.60 | 9.19E-03 |
| VYZT01054231.1_28659 | upstream10k | -5397 | Tbx3 | 1.14 | -0.04 | 1.09 | 1.79E-62 |
| VYZT01015202.1_12345 | upstream10k | -7884 | Tesc | 4.22 | 0.27 | 4.48 | 2.09E-21 |
| VYZT01043817.1_79371 | upstream10k | -3613 | Timp2 | -2.27 | 3.12 | 0.84 | 2.17E-25 |
| VYZT01065593.1_63296 | upstream10k | -4279 | Tjap1 | 1.89 | 0.60 | 2.48 | 6.65E-03 |
| VYZT01048608.1_145234 | upstream10k | -9131 | Tmem132e | -2.42 | 3.15 | 0.74 | 8.97E-05 |
| VYZT01038349.1_51338 | upstream10k | -3383 | Tmem150c | 2.55 | 0.55 | 3.10 | 7.39E-06 |
| VYZT01055017.1_26135 | upstream10k | -3649 | Tnfrsf1a | 1.22 | -0.14 | 1.08 | 3.55E-06 |
| VYZT01056195.1_217295 | upstream10k | -5041 | Tsnax | -2.21 | 3.01 | 0.80 | 9.62E-04 |
| VYZT01013226.1_4245 | upstream10k | -9880 | Uggt1 | 2.21 | -0.05 | 2.16 | 8.33E-03 |
| VYZT01016526.1_27452 | upstream10k | -8833 | Vdac2 | -1.13 | 0.97 | -0.16 | 0.00E+00 |
| VYZT01062051.1_93519 | upstream10k | -6568 | Veph1 | -2.37 | 3.22 | 0.84 | 6.37E-04 |
| VYZT01054186.1_25395 | upstream10k | -3481 | Wrap73 | 1.49 | 0.23 | 1.72 | 9.30E-03 |
| VYZT01054186.1_25390 | upstream10k | -3476 | Wrap73 | 1.70 | -0.32 | 1.38 | 7.25E-03 |
| VYZT01015404.1_13171 | upstream10k | -2279 | Xirp1 | 2.02 | -0.01 | 2.01 | 0.00E+00 |
| VYZT01065369.1_129587 | upstream10k | 3343 | Ywhab | 2.23 | 0.59 | 2.82 | 3.92E-04 |
| VYZT01029293.1_324135 | upstream10k | -8798 | Zbtb16a | -5.78 | 6.19 | 0.41 | 3.10E-94 |
| VYZT01019555.1_43718 | upstream10k | -3075 | Znf521 | 1.96 | 0.53 | 2.49 | 8.94E-03 |
| VYZT01034606.1_36379 | upstream10k | -3134 | Znf831 | 1.86 | 0.64 | 2.50 | 6.54E-04 |
| VYZT01007738.1_90317 | downstream10k | 30721 | Abtb1 | 2.15 | 0.35 | 2.49 | 8.42E-04 |
| VYZT01015144.1_43738 | downstream10k | 2382 | Acvr1b | 1.91 | 0.31 | 2.22 | 5.75E-03 |
| VYZT01011226.1_138207 | downstream10k | 3794 | Adcyap1 | 2.03 | 0.78 | 2.82 | 8.07E-03 |
| VYZT01026412.1_270170 | downstream10k | 13379 | Amz1 | 1.16 | -0.64 | 0.53 | 1.32E-183 |
| VYZT01028038.1_155097 | downstream10k | 7657 | Ankrd1 | 1.34 | 0.23 | 1.57 | 8.15E-03 |
| VYZT01041765.1_296286 | downstream10k | 9960 | Ap1s3 | -1.92 | 2.30 | 0.37 | 0.00E+00 |
| VYZT01006840.1_1185 | downstream10k | 2478 | Apc2 | 2.79 | 0.89 | 3.67 | 6.28E-05 |
| VYZT01052816.1_18186 | downstream10k | 7088 | Arf6 | 2.08 | 0.81 | 2.89 | 7.65E-03 |
| VYZT01050018.1_42552 | downstream10k | 21414 | Azin1 | 2.23 | 0.35 | 2.58 | 0.00E+00 |
| VYZT01044410.1_35996 | downstream10k | 10612 | Bhlhe40 | -2.93 | 3.04 | 0.11 | 4.06E-62 |
| VYZT01035958.1_430845 | downstream10k | 4842 | Bid | 1.70 | 0.38 | 2.08 | 1.15E-10 |
| VYZT01016176.1_154335 | downstream10k | 4759 | Bnip3l | -2.14 | 2.89 | 0.74 | 1.47E-03 |
| VYZT01027453.1_22324 | downstream10k | 8586 | Ca216 | 2.08 | 0.73 | 2.81 | 9.19E-03 |
| VYZT01017233.1_39875 | downstream10k | 1632 | Cacfd1 | 2.25 | -1.10 | 1.15 | 5.71E-03 |
| VYZT01034731.1_62787 | downstream10k | 3437 | Ccr7 | 1.81 | -0.47 | 1.34 | 1.07E-209 |
| VYZT01005345.1_209473 | downstream10k | 11845 | Cdc42 | 2.21 | 0.81 | 3.02 | 1.76E-03 |
| VYZT01021257.1_10509 | downstream10k | 8195 | Cebpb | 2.70 | 0.67 | 3.37 | 0.00E+00 |
| VYZT01032656.1_19277 | downstream10k | 621 | Cenpx | 2.16 | 0.82 | 2.97 | 3.29E-04 |
| VYZT01041670.1_79715 | downstream10k | -10008 | Clec3a | -2.72 | 3.47 | 0.75 | 2.10E-12 |
| VYZT01030594.1_8910 | downstream10k | 4225 | Cspg5 | 2.22 | 0.20 | 2.42 | 4.21E-03 |
| VYZT01041617.1_72835 | downstream10k | 3356 | Dnah1 | 1.67 | -0.14 | 1.53 | 7.47E-03 |
| VYZT01053475.1_10521 | downstream10k | 8097 | Dock1 | 2.07 | 0.67 | 2.74 | 5.32E-03 |
| VYZT01019836.1_40664 | downstream10k | 1453 | Dusp14 | -2.25 | 3.12 | 0.87 | 1.60E-05 |
| VYZT01036810.1_2489 | downstream10k | 10682 | Edn2 | -2.50 | 2.62 | 0.11 | 3.06E-17 |
| VYZT01022256.1_161896 | downstream10k | 15311 | Endod1 | 1.32 | 0.05 | 1.37 | 4.29E-07 |
| VYZT01044188.1_49835 | downstream10k | -11869 | Evpl | -2.13 | 2.41 | 0.28 | 2.90E-03 |
| VYZT01058793.1_74090 | downstream10k | 12827 | Fbxo41 | 1.88 | 0.62 | 2.50 | 6.57E-03 |
| VYZT01029965.1_45016 | downstream10k | 9693 | Fundc1 | 4.55 | -0.69 | 3.86 | 1.87E-03 |
| VYZT01031530.1_87953 | downstream10k | -12832 | Galnt16 | 2.04 | 0.68 | 2.72 | 9.34E-03 |
| VYZT01008397.1_50399 | downstream10k | 49269 | Grin2a | 2.11 | -1.20 | 0.91 | 4.45E-03 |
| VYZT01005806.1_5091 | downstream10k | 3803 | Grm4 | -2.40 | 2.83 | 0.43 | 3.46E-04 |
| VYZT01034503.1_356973 | downstream10k | 6954 | Grxcr2 | 2.46 | -0.16 | 2.30 | 3.15E-11 |
| VYZT01030555.1_147833 | downstream10k | 9135 | Hsd11b1l | 2.17 | 0.78 | 2.95 | 1.08E-04 |
| VYZT01004871.1_434577 | downstream10k | 5065 | Il1rap | -1.94 | 2.73 | 0.79 | 7.26E-03 |
| VYZT01033104.1_29831 | downstream10k | 17227 | Kank4 | 1.78 | 0.00 | 1.78 | 1.37E-03 |
| VYZT01007587.1_23264 | downstream10k | 4975 | Kcna3 | 1.04 | -0.36 | 0.68 | 5.11E-13 |
| VYZT01009861.1_63655 | downstream10k | 11304 | Kcnc4 | 1.87 | 0.19 | 2.06 | 7.38E-03 |
| VYZT01049467.1_5557 | downstream10k | 7337 | Kcne1 | 2.02 | -0.19 | 1.83 | 8.49E-03 |
| VYZT01024114.1_57730 | downstream10k | 17581 | Kcnj5 | 1.33 | -0.55 | 0.77 | 2.02E-04 |
| VYZT01002114.1_104141 | downstream10k | 78204 | Lrrtm3 | 2.44 | 0.13 | 2.57 | 1.61E-03 |
| VYZT01020495.1_18462 | downstream10k | 4744 | Ly6e | 1.85 | 0.71 | 2.56 | 9.76E-03 |
| VYZT01050182.1_65463 | downstream10k | 10222 | Mgat5b | 2.13 | 0.79 | 2.91 | 3.77E-03 |
| VYZT01043210.1_377112 | downstream10k | 3879 | Mmp15 | 2.25 | 0.16 | 2.42 | 1.44E-04 |
| VYZT01038971.1_67758 | downstream10k | 14040 | Mtmr3 | 1.96 | 0.65 | 2.60 | 7.97E-03 |
| VYZT01028657.1_113852 | downstream10k | 28815 | Mtmr4 | 2.73 | 0.75 | 3.48 | 5.21E-05 |
| VYZT01063190.1_73792 | downstream10k | 2544 | Ncoa6 | 2.16 | 0.78 | 2.94 | 1.96E-03 |
| VYZT01009701.1_357617 | downstream10k | 9764 | Npy | 2.07 | 0.79 | 2.85 | 7.43E-03 |
| VYZT01013229.1_67324 | downstream10k | 8224 | Olig2 | 1.93 | 0.76 | 2.68 | 3.65E-03 |
| VYZT01034516.1_197862 | downstream10k | 6762 | Osgin1 | 1.56 | -0.06 | 1.49 | 8.12E-03 |
| VYZT01030775.1_8862 | downstream10k | 8366 | Otof | 1.84 | 0.56 | 2.41 | 6.46E-03 |
| VYZT01022943.1_126396 | downstream10k | 1864 | Pam16a | 1.80 | -0.11 | 1.68 | 7.26E-03 |
| VYZT01049438.1_111634 | downstream10k | 6626 | Parp6 | 1.48 | 0.45 | 1.93 | 9.90E-04 |
| VYZT01021899.1_157794 | downstream10k | 7557 | Pgpep1 | 1.56 | 0.38 | 1.93 | 4.78E-03 |
| VYZT01033199.1_37789 | downstream10k | 10160 | Phc3 | 2.63 | 0.89 | 3.52 | 5.00E-05 |
| VYZT01001927.1_32856 | downstream10k | 8096 | Plekhf2 | 1.82 | 0.22 | 2.04 | 8.65E-03 |
| VYZT01006666.1_5284 | downstream10k | 16263 | Pm20d1 | 2.21 | 0.49 | 2.70 | 1.70E-04 |
| VYZT01062145.1_11303 | downstream10k | 9683 | Prorsd1 | 1.62 | 0.41 | 2.03 | 6.25E-91 |
| VYZT01030594.1_67439 | downstream10k | 19136 | Ptpn23 | 2.65 | 0.87 | 3.52 | 2.53E-04 |
| VYZT01060310.1_142045 | downstream10k | 14366 | Rcn2 | -1.80 | 2.22 | 0.42 | 4.32E-03 |
| VYZT01058133.1_579543 | downstream10k | 9761 | Rhbgb | -1.44 | 1.89 | 0.45 | 7.14E-04 |
| VYZT01019953.1_70525 | downstream10k | 18741 | Rnd2 | 2.78 | 0.34 | 3.12 | 1.09E-94 |
| VYZT01005938.1_102769 | downstream10k | 9570 | Rnpep | 2.59 | 0.69 | 3.28 | 1.42E-04 |
| VYZT01016446.1_119486 | downstream10k | 6179 | Rp1l1 | 1.45 | 0.17 | 1.62 | 3.44E-07 |
| VYZT01035271.1_122597 | downstream10k | 44240 | Rtkn2 | 2.19 | -0.17 | 2.03 | 1.02E-03 |
| VYZT01039064.1_421522 | downstream10k | 106806 | Satb2 | 2.29 | 0.46 | 2.75 | 7.45E-04 |
| VYZT01001272.1_165023 | downstream10k | 12420 | Scarb1 | 1.75 | 0.44 | 2.20 | 0.00E+00 |
| VYZT01021211.1_176908 | downstream10k | -16785 | Sertad4 | -1.76 | 2.16 | 0.39 | 6.66E-03 |
| VYZT01009668.1_76179 | downstream10k | 3731 | Sfn | -2.24 | 3.10 | 0.86 | 1.16E-03 |
| VYZT01050763.1_55249 | downstream10k | 2588 | Sigirr | 2.17 | 0.62 | 2.78 | 5.27E-04 |
| VYZT01027181.1_75376 | downstream10k | 26992 | Slc25a24 | 2.01 | 0.75 | 2.76 | 8.29E-03 |
| VYZT01051570.1_17968 | downstream10k | 4128 | Smarca4 | 2.10 | 0.74 | 2.84 | 8.98E-04 |
| VYZT01013499.1_6260 | downstream10k | 33087 | Smg1 | 2.67 | 0.37 | 3.04 | 1.86E-42 |
| VYZT01053683.1_143726 | downstream10k | 70898 | Specc1 | 1.28 | -0.43 | 0.85 | 0.00E+00 |
| VYZT01048233.1_541678 | downstream10k | 28625 | Spopl | 1.92 | 0.68 | 2.60 | 1.53E-03 |
| VYZT01022883.1_3735 | downstream10k | 14896 | Srsf11 | 2.04 | 0.38 | 2.42 | 6.00E-03 |
| VYZT01063294.1_60384 | downstream10k | 31410 | Ssh1 | 1.23 | -0.25 | 0.97 | 1.30E-54 |
| VYZT01060117.1_376500 | downstream10k | 37604 | St8sia2 | -2.55 | 3.49 | 0.94 | 1.83E-04 |
| VYZT01060714.1_65432 | downstream10k | 1555 | Tbxa2r | 1.97 | 0.79 | 2.77 | 6.66E-03 |
| VYZT01003536.1_36687 | downstream10k | 11510 | Timm44 | 1.43 | 0.14 | 1.57 | 4.90E-03 |
| VYZT01037739.1_48773 | downstream10k | 6337 | Tipin | 2.06 | 0.76 | 2.82 | 6.48E-03 |
| VYZT01012434.1_624428 | downstream10k | 4210 | Tmem11 | 2.52 | -2.00 | 0.52 | 2.55E-03 |
| VYZT01032542.1_90611 | downstream10k | 3098 | Tmem140 | -2.14 | 2.86 | 0.72 | 3.49E-03 |
| VYZT01034751.1_2988 | downstream10k | 3607 | Tnfrsf25 | 1.91 | 0.66 | 2.57 | 2.61E-03 |
| VYZT01063263.1_1410 | downstream10k | 20086 | Ubash3b | -2.94 | 3.63 | 0.69 | 1.48E-05 |
| VYZT01005432.1_14697 | downstream10k | 23910 | Vwc2l | 2.09 | 0.59 | 2.68 | 4.04E-03 |
| VYZT01015256.1_64442 | downstream10k | 8120 | Wasf3 | 2.11 | 0.72 | 2.83 | 4.92E-04 |
| VYZT01034636.1_110924 | downstream10k | 13563 | Wdyhv1 | 2.15 | -0.17 | 1.97 | 6.57E-03 |
| VYZT01042238.1_32338 | downstream10k | 29297 | Xkr6 | 2.56 | 0.40 | 2.96 | 8.63E-05 |
| VYZT01008648.1_7505 | downstream10k | 7207 | Zdhhc15b | 1.75 | -0.31 | 1.44 | 4.17E-03 |
